# From Rings to Properties: Understanding the Effect of Annelation on Pyrene

**DOI:** 10.1021/acs.joc.5c01401

**Published:** 2025-08-29

**Authors:** Alexandra Wahab, Renana Gershoni-Poranne

**Affiliations:** † Laboratory for Organic Chemistry, Department of Chemistry and Applied Biosciences, 31064 ETH Zurich, Zurich 8093, Switzerland; ‡ Schulich Faculty of Chemistry and the Resnick Sustainability Center for Catalysis, 26747 Technion - Israel Institute of Technology, Haifa 32000, Israel

## Abstract

Pyrene is a central building block in organic materials chemistry, valued for its rigid aromatic core, high fluorescence, and rich capacity for structural elaboration. However, the fundamental relationship between its annelation pattern and resulting electronic properties remains underexplored. In this work, we present a comprehensive computational study of 4,766 pyrene-based polybenzenoid hydrocarbons from the COMPAS-3D data set. By systematically categorizing annelation patterns based on the positions of fused benzene rings, we uncover clear structure–property trends governing key electronic parameters, including molecular orbital energies and oxidation and reduction potentials. We find that annelations at different positions exert different effects, which combine additively, and that these trends correlate with the distribution of fixed versus migrating Clar sextets. We further show that extended linear annelation can dominate electronic behavior, superseding local pyrene effects. Additionally, we evaluate the effect of torsional strain, revealing a clear link between annelation patterns and thermodynamic molecular destabilization. These insights provide a framework for the rational design of functional pyrene-based molecules.

## Graphical abstract

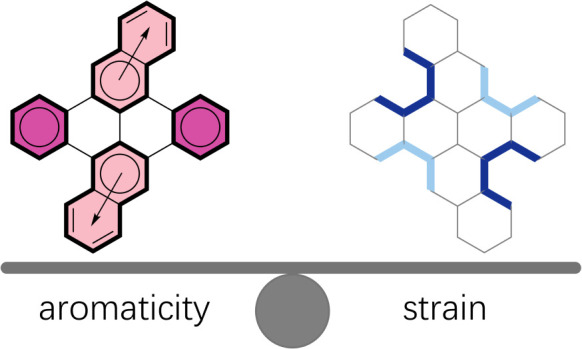

## 1 Introduction

Polycyclic aromatic systems (PASs) are central to a broad range of functional molecules, particularly with applications in organic electronics and optoelectronics.^1–5^ Over the past several years, we have undertaken a systematic effort to chart the chemical space of PASs through the COMPAS (COMputational database of PASs) Project^6–9^ —an evolving suite of curated data sets designed to support data-driven investigations of aromatic systems. These studies have revealed how features such as linear annulation and geometric motifs shape key molecular properties^10–12^ and have enabled predictive models for rational molecular design.^13^

As with other PASs, pyrene-based molecules are used extensively in organic (opto)­electronics,^14–17^ including for blue light emitting diodes^18,19^ and luminescent materials.^20^ However, among the many PAS scaffolds, pyrene holds a special place, as it plays a key role in many other functionalities. As the smallest Kekuléan *peri*-condensed polybenzenoid hydrocarbon (PBH), pyrene also plays a central role in host–guest chemistry and has even been called the “guest of honor”.^21^ Thanks to its high fluorescence, pyrene is also widely used as a chemosensor and a biosensor.^22–25^ Additionally, pyrene-based supramolecular structures, such as metallocycles and cages,^26^ as well as covalent-^27^ and metal–organic frameworks,^28–30^ have been used for photocatalysis.

Despite this widespread use, the relationship between structural variation and electronic behavior in pyrene-based systems remains incompletely understood. Previous studies have investigated various aspects of structure modification, such as the regioisomerism of donor–acceptor groups,^31^ the effect of combining an aza group^27^ or a helicene,^32^ and other methods of functionalization.^33,34^ However, a general understanding of the effect of benzannelation on the pyrene core has, to the best of our knowledge, not yet been developed.

To address this gap, we present a data-driven computational investigation of pyrene-based PBHs, aimed at revealing how different annelation motifs modulate molecular properties. Our focus on this restricted chemical space is motivated by several considerations. First, PBHs constitute a chemically relevant and synthetically tractable subclass of PASs, encompassing many well-studied materials in organic electronics and materials (*vide supra*). Even for these supposedly simple systems, it has been shown by us and others^35–37^ that benzannelation can have a profound effect on molecular properties. Second, by excluding heteroatoms and substituents, we isolate the influence of core conjugation geometry on electronic properties, enabling a clearer interpretation of structure–property trends without the confounding effects of electronic perturbations. Third, the benzenoid chemical space, though combinatorially rich, remains finite and well-defined, making it amenable to systematic and exhaustive analysis.

We explore the complete chemical space of pyrene-based PBHs, in which the nonpyrene components are purely *cata*-condensed, comprising from 5 to 10 benzene rings (5 ≤ *n*_rings_ ≤ 10), analyzing their electronic and structural properties via quantum chemical calculations. Our investigation provides a deeper understanding of the behavior of pyrene-based PBHs, revealing underlying structure–property relationships and providing principles for rational design of functional materials for (opto)­electronic applications.

## 2 Results and Discussion

Pyrene contains two distinct ring types, as shown in Figure 1A: the *a* rings, which are each fused to two other rings, and the *b* rings, which are fused to three other rings. This classification not only captures the inherent distinction between the two types of benzene rings of the pyrene core, but also allows us to systematically categorize patterns based on easily identifiable structural features. Thus, we classified each of the 4,766 molecules in our data set according to the annelation patterns onto the *a* and *b* rings. Figure 1B depicts the eight possible annelation patterns: **a**, **b**, **aa**, **bb**, **ab**, **aab**, **abb**, **aabb** (further details on the classification and an overview of the data are provided in Section S2 of the Supporting Information).

**Figure 1:**
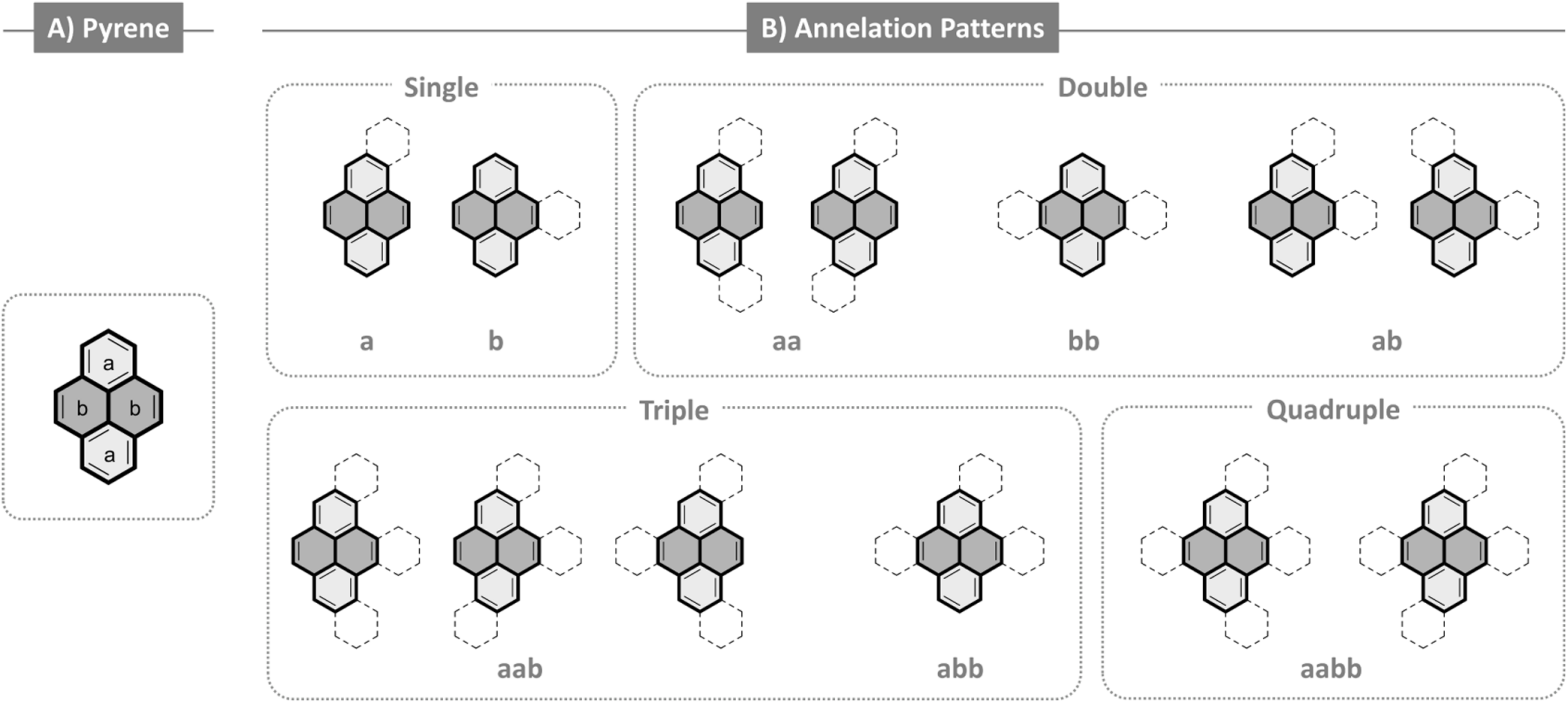
A) Structure of pyrene with *a* and *b* rings denoted. B) The eight annelation patterns defined in our classification scheme, separated by the number of annelations on the pyrene core, from single to quadruple. In all structures, *a* rings are colored light gray and *b* rings are colored dark gray.

To explore the effects of annelation position, we investigated the relationship between the eight patterns and six different molecular properties: energy of the highest occupied molecular orbital (HOMO), energy of the lowest unoccupied molecular orbital (LUMO), the HOMO–LUMO energy gap (Δ*E*_H–L_), adiabatic ionization potential (aIP), adiabatic electron affinity (aEA), and relative energy (*E*_rel_). The relative energy was calculated as the difference in energy between a given structure and its lowest-energy isomer.

Plotting the various property distributions for each group (Figure 2), we observed a certain regularity: when arranging the distributions such that the mean values appear in a monotonic trend (descending for HOMO; ascending for LUMO, Δ*E*_H‑L_, aIP, and aEA), the annelation patterns always appear in the same order—**aa**, **aab**, **a**, **aabb**, **ab**, **abb**, **b**, **bb**. Only *E*_rel_ does not follow the same order.

**Figure 2:**
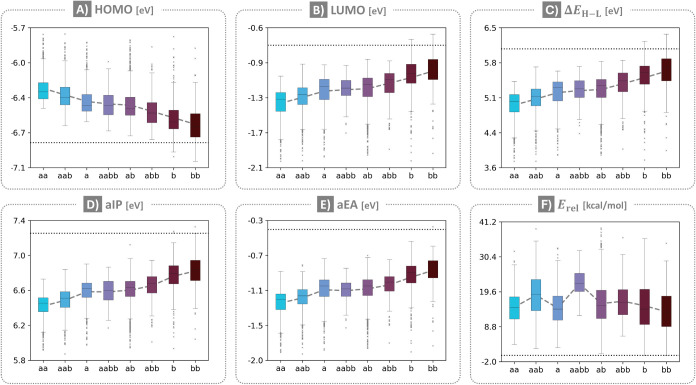
Box-and-whisker plots of the DFT-calculated properties colored by annelation pattern: A) HOMO; B) LUMO; C) Δ*E*_H‑L_; D) aIP; E) aEA; F) *E*_rel_. The dashed gray lines indicate the average value, the dotted black lines indicate the value of the parent pyrene.

Two important conclusions were drawn from this simple observation. First, that our simple strategy of classifying the molecules provides an entry point for investigating the relationship between properties and annelation patterns. At the same time, we note that the trends reflect the average values for each group; the significant overlap between groups suggests that the annelation pattern itself is not sufficient for determining properties uniquely. Second, that the first five molecular properties are likely governed by similar effects, whereas the *E*_rel_ is determined by other (or additional) factors. Indeed, our previous work on cc-PBHs demonstrated that the first five properties are dominated by the number of linearly annelated rings, while the sixth shows great sensitivity to geometric distortion,^6^ in particular to the torsional strain introduced by nonplanar motifs (e.g., helices).^11,12^

Therefore, we considered the annelation patterns as a conceptual framework for exploring the structure–property relationships in pyrene-based PBHs. Based on the similarities noted above and our previous work, we consider the first five properties to be distinct from the sixth property, and analyze them separately.

### 2.1 HOMO, LUMO, Δ*E*_H‑L_, aIP, aEA

In this section, we investigate the trends observed for the first five properties presented in Figure 2, analyze the effects of *a*-annelation versus *b*-annelation, and offer a resonance structure (RS)-based rationalization for the conserved order of annelation patterns. Finally, we place these insights into the broader context of cc-PBH structure–property relationships that we reported in earlier work.

#### 2.1.1 Effect of *a*- and *b*-Annelation

As a starting point, we first considered the simplest metric—the total number of annelations. As can be seen from the order of the groups in Figure 2, the increase/decrease in the average property values does not correspond to fewer/more annelation sites. Meaning, the overall number of annelations is not the determining factor; rather, the specific location(s) of annelation play a key role. We further verified that the observed trends are not simply a result of increasing molecular size (see Section S3 in the Supporting Information).

Closer inspection revealed that *a*-annelation has an opposite effect to *b*-annelation: the **aa** group shows the highest average HOMO value and the lowest average LUMO, Δ*E*_H–L_, aIP, and aEA values, while the **bb** group shows the opposite. We also observed that, in this ordering, **aa** ranks lower than **a** and **bb** ranks higher than **b**, which indicates that the effects are cumulative. Furthermore, the **ab** and **aabb** groups are located in the middle of the distributions, demonstrating that when the two annelation types are present together, they cancel each other out. In fact, a semiquantitative estimation of the relative strengths of the *a*-annelation versus *b*-annelation effects can be made. If the magnitudes of the effects were identical, then the pair **a**/**b** and the pair **aab**/**abb** would be symmetrically spaced around the center of the distribution. Yet, we find **aab** between **aa** and **a**, and **abb** between **ab** and **b**. Additionally, **aabb** and **ab** are not identical, with **aabb** placed closer to the **a** side of the spectrum. All of these observations suggest that the effect of *a*-annelation is stronger than that of *b*-annelation.

#### 2.1.2 Rationalization with Resonance Structures

Recent work from our group demonstrated that resonance structures (RSs) can provide an intuitive explanation for PAS behavior,^9^ hence we chose to employ them in this investigation. For each of the eight annelation patterns, we drew the Clar structure—the RS containing the largest number of Clar sextets^38–40^ (disjoint sets of 6π electrons, commonly denoted with a circle). The Clar structure is expected to be the most contributing RS and therefore the most significant for determining the properties of a given molecule. For some molecules, more than one Clar structure can be drawn. We denote these with the conventional arrow indicating a *migrating* Clar sextet (Figure 3).

**Figure 3:**
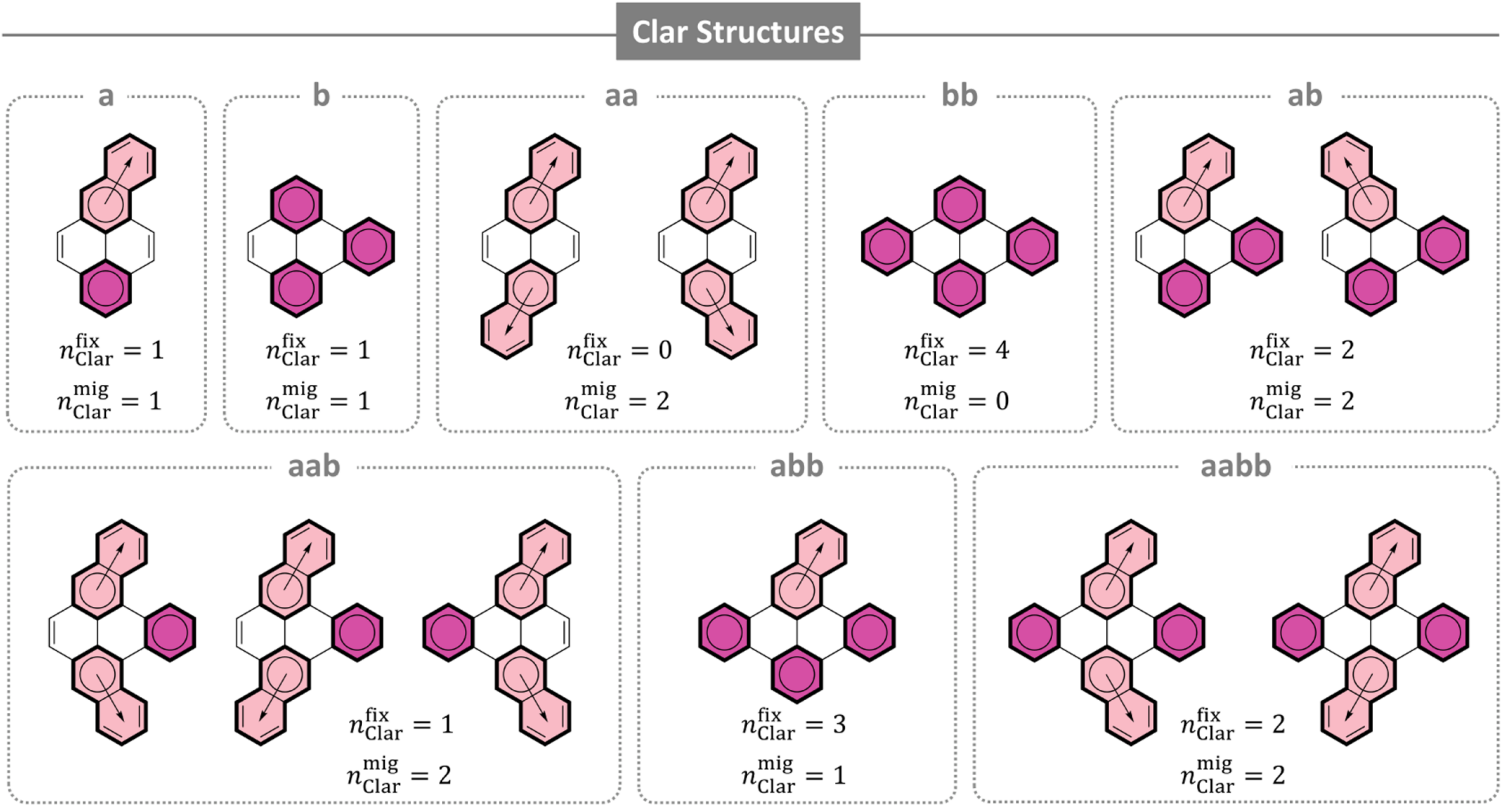
RSs representing each annelation pattern, with $n_{\mathrm{Clar}}^{\mathrm{fix}}$ and $n_{\mathrm{Clar}}^{\mathrm{mig}}$ detailed. Clar sextets are denoted with a circle. Rings containing fixed Clar sextets are colored in dark pink; rings containing migrating Clar sextets are colored in light pink, with arrows indicating the migration.

The number of Clar sextets often corresponds to stability, absorption energies, and Δ*E*_H–L_ values of PBHs.^41,42^ In general, as the number of Clar sextets increases, the molecule is considered “more aromatic”, is more thermodynamically stable, and has a larger Δ*E*_H–L_. Therefore, we considered it a natural approach to simply examine the relationship between the total number of Clar sextets in a molecule, $n_{\mathrm{Clar}}^{\mathrm{tot}}$ , and the annelation pattern order. Somewhat to our surprise, we observed no clear correlation between $n_{\mathrm{Clar}}^{\mathrm{tot}}$ (Table 1, first column) and the pattern order.

**Table 1:** $n_{\mathrm{Clar}}^{\mathrm{tot}}$ , $n_{\mathrm{Clar}}^{\mathrm{fix}}$ , $n_{\mathrm{Clar}}^{\mathrm{mig}}$ , and ΔClar Values for Each Annelation Pattern (Arranged in Order from Lowest to Highest Δ*E*_H–L_)

Pattern	$n_{\mathrm{Clar}}^{\mathrm{tot}}$	$n_{\mathrm{Clar}}^{\mathrm{fix}}$	$n_{\mathrm{Clar}}^{\mathrm{mig}}$	ΔClar
**aa**	2	0	2	–2
**aab**	3	1	2	–1
**a**	2	1	1	0
**aabb**	4	2	2	0
**ab**	3	2	1	1
**abb**	3	3	1	2
**b**	3	3	0	3
**bb**	4	4	0	4

Counting *fixed* and *migrating* Clar sextets separately ( $n_{\mathrm{Clar}}^{\mathrm{fix}}$ and $n_{\mathrm{Clar}}^{\mathrm{mig}}$ , respectively), however, proved a more successful approach. As shown in Table 1, $n_{\mathrm{Clar}}^{\mathrm{fix}}$ rises steadily along the pattern order, our first indication of a potential relationship. In contrast, $n_{\mathrm{Clar}}^{\mathrm{mig}}$ does not show any regularities. In fact, $n_{\mathrm{Clar}}^{\mathrm{mig}}$ appears to have a deleterious effect: **abb** and **b** both have $n_{\mathrm{Clar}}^{\mathrm{fix}}$ = 3, but **abb** is lower in the order; similarly, **aabb** and **ab** both have $n_{\mathrm{Clar}}^{\mathrm{fix}}$ = 2, but **aabb** is lower in the order. In both cases, the lower group has a higher $n_{\mathrm{Clar}}^{\mathrm{mig}}$ value. Based on this, we defined a new metric, $\Delta \mathrm{Clar}=n_{\mathrm{Clar}}^{\mathrm{fix}}-n_{\mathrm{Clar}}^{\mathrm{mig}}$ (Table 1, fourth column). Gratifyingly, ΔClar shows a clear and steady increase along the pattern order, suggesting that it is related to these molecular properties. To verify this, we examined the quantitative relationship between ΔClar and the five molecular properties. In Figure 4A, we plot the mean value of each property against the ΔClar values and find remarkable agreements in all cases.

**Figure 4:**
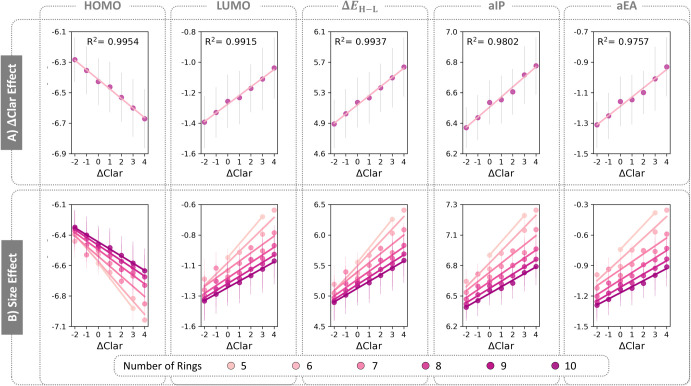
Relationship between ΔClar and molecular properties for HOMO, LUMO, Δ*E*_H‑L_, aIP, aEA. A) Scatter plots of mean property value against ΔClar. The calculated regression line is shown in light pink. B) Scatter plots of mean property value against ΔClar, separated by *n*_rings_. The calculated regression line for each *n*_rings_ subgroup is shown in the same color-coding. For both panels: all energies reported in eV; the standard deviation for each data point is indicated by a vertical line.

Separating the data according to *n*_rings_ (Figure 4B) reveals an interesting relationship with molecular size, although ΔClar itself is not a size-dependent metric (see Section S3 of the Supporting Information). ΔClar shows a strong correlation with properties for each individual value of *n*_rings_, but the slopes become more pronounced in smaller molecules. Meaning, the dependence on ΔClar is more dramatic in smaller molecules, likely because the pyrene core comprises a greater component of the complete structure. The trends also reveal that molecules with higher ΔClar have greater differentiation by size. This means that certain annelation patterns (e.g., **b** and **bb**) provide greater flexibility in tailoring molecular properties via extended annelation.

Thus, the RS-analysis revealed that fixed Clar sextets contribute in the “expected” way, i.e., increasing $n_{\mathrm{Clar}}^{\mathrm{fix}}$ lowers the HOMO and raises the LUMO (e.g., group **bb**), whereas migrating Clar sextets counteract this effect (e.g., group **aa**). Because each migrating Clar replaces a fixed Clar in the original pyrene core, we hypothesize that it is not that the migrating Clar has an opposite effect, but rather that the effect (i.e., aromaticity) of a fixed sextet is greater than that of a migrating one. This may seem counterintuitive, as migrating Clar sextets imply more RSs and therefore greater electron delocalization. However, Clar’s rule favors fixed disjoint aromatic sextets; migrating Clar sextets reflect the inability to localize sextets on specific rings across all RSs. This indicates that no ring enjoys the full benefit of a Clar sextet. We note that this explanation aligns with previous work by Randić, which also found that migrating Clar sextets have weaker aromaticity than fixed ones.^43^

#### 2.1.3 Pyrene Cores and Linear Stretches

We next sought to combine these new insights with our existing knowledge. We previously demonstrated that the longest stretch of consecutive linearly annelated benzenes (the “Longest L”) is the dominant motif in determining the same five properties in purely *cata*-condensed PBHs (cc-PBHs).^6,11^ Thus, two questions arose: a) *Can the two classification types be combined for PBHs containing both substructures?* and b) *If so, which substructure plays the dominant role?*

To answer these questions, we generated box plots of the Δ*E*_H–L_ values for the entire pyrene data set, separated by their annelation pattern and further subdivided according to the number of rings in the longest linear stretch, *n*_LL_ (Figure 5). As discussed before, the mean Δ*E*_H–L_ value increases as the ratio of *b*-annelation to *a*-annelation increases. However, this new subdivision of data also revealed the source of the broad distribution of Δ*E*_H–L_ values within each annelation pattern, with higher *n*_LL_ values corresponding to lower Δ*E*_H–L_, just as in cc-PBHs. Thus, the answer to our first question is: yes, it is possible to combine the two effects in molecules containing both a pyrene core and a linear component.

**Figure 5:**
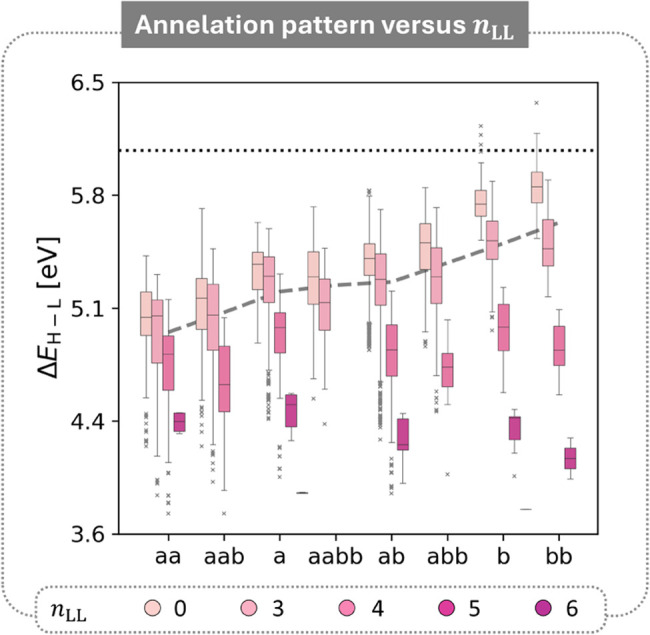
Box-and-whisker plots of the Δ*E*_H–L_ distributions, separated by annelation pattern and further subdivided and colored by *n*_LL_.

To answer the second question, we closely examined the trends of each of the features within each group. We observed that the *n*_LL_ effect becomes more noticeable as the number of *b*-annelation increases, and is most pronounced in the **b** and **bb** groups. In contrast, the **a** and **aa** groups show a strong effect only for *n*_LL_ ≥ 4. This suggests that the balance between the pyrene core and the linear stretch is pattern-dependent. Regardless, we note that for all annelation patterns, the dark pink and magenta boxes (higher *n*_LL_ values) cover similar ranges. This implies that the annelation pattern effect is overpowered by the Longest L effect when the linear stretch is long enough. We note that for the **aabb** group, we do not have data for *n*_LL_ > 3, because the molecules contained in COMPAS-3D are too small to have such long linear stretches in addition to the quadruply annelated pyrene.

We used the ΔClar metric again, this time to compare between the annelation patterns and *n*_LL_. In Figure 6 we present scatter plots of the five molecular properties against ΔClar, where the data have been grouped by *n*_LL_. In comparing the plots, it is clear that lower *n*_LL_ values (light pink colors) show a stronger agreement with ΔClar, whereas higher *n*_LL_ values (darker pink colors) show a weakening of the agreement and even a reversal of the trend. This, therefore, corroborates that *n*_LL_ overpowers the pyrene annelation pattern when the stretch becomes long enough. It further demonstrates that different patterns have different susceptibility to linear stretches. For example, the patterns with low ΔClar (e.g., **a** and **aa**) show relatively little change in property value ranges despite large changes to the linear stretch, while those with higher ΔClar show much more dramatic variation in molecular property range. Once again, this presents possibilities for tuning properties via annelation.

**Figure 6:**
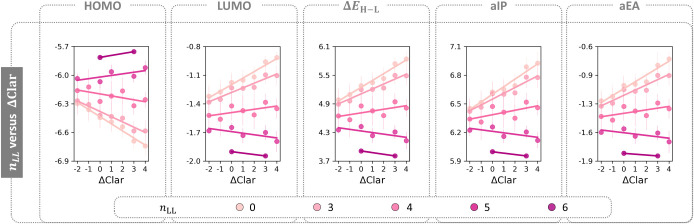
Scatter plots of the average Δ*E*_H–L_ for each ΔClar value, further subdivided and colored by *n*_LL_. The standard deviation for each data point is indicated by a vertical line. The calculated regression line for each *n*_LL_ subgroup is shown in the same color-coding.

### 2.2 Relative Energy (*E*_rel_)

As opposed to the previous five properties, the order observed for *E*_rel_ is **bb** < **a** < **aa** < **b** < **ab** < **abb** < **aab** < **aabb**. We hypothesized that this is because *E*_rel_ comprises two separate contributions: a) conjugation (e.g., stabilization via delocalization and aromaticity) and b) geometric distortion/strain. The effects of conjugation on *E*_rel_ are likely similar to those described above for the other properties, meaning they are related to the number of Clar sextets. In contrast, the effects of strain require a separate method of evaluation. Therefore, we first implemented an approach to quantify the strain in the various annelation patterns. Then, we defined simple metrics to allow us to identify the underlying structure–property relationship. Finally, we combined the insights pertaining to strain with the overall *E*_rel_ of the pyrene-based PBHs.

#### 2.2.1 Quantification of Strain

To evaluate the strain in different PBHs, we require an unstrained reference system. A common approach is to use a minimally strained isomer as the reference and construct a homodesmotic/isodesmic reaction. In other words, to calculate the difference in energy between a given molecule and a PBH with the same *n*_rings_, in which the rings are arranged differently so as to have minimal/no strain. However, changing the arrangement of rings also changes the number of Clar sextets, which we already established has a strong influence on the molecular properties. Therefore, to quantify the strain, we adopted instead the ″frozen-core″ approach (illustrated in Figure 7A) that we previously applied successfully to phenylene molecules.^44^ To do so, we curated a test set, which includes every possible structure that can be generated by having a maximum of one annelated ring at each of the *a* and *b* positions (a total of 13 molecules; Figure 7B). The molecules are named after the pattern they belong to with an added superscript “s*x*” (where “s” stands for “sample” and “*x*” is a running integer) to distinguish between isomers belonging to the same patterns (e.g., there are two **aa** isomers, denoted **aa** ^**s1**^ and **aa** ^**s2**^, respectively). This distinction is also important because every name corresponds to a specific molecule, whereas in the pattern nomenclature, each name represents all molecules with the same annelation pattern.

**Figure 7:**
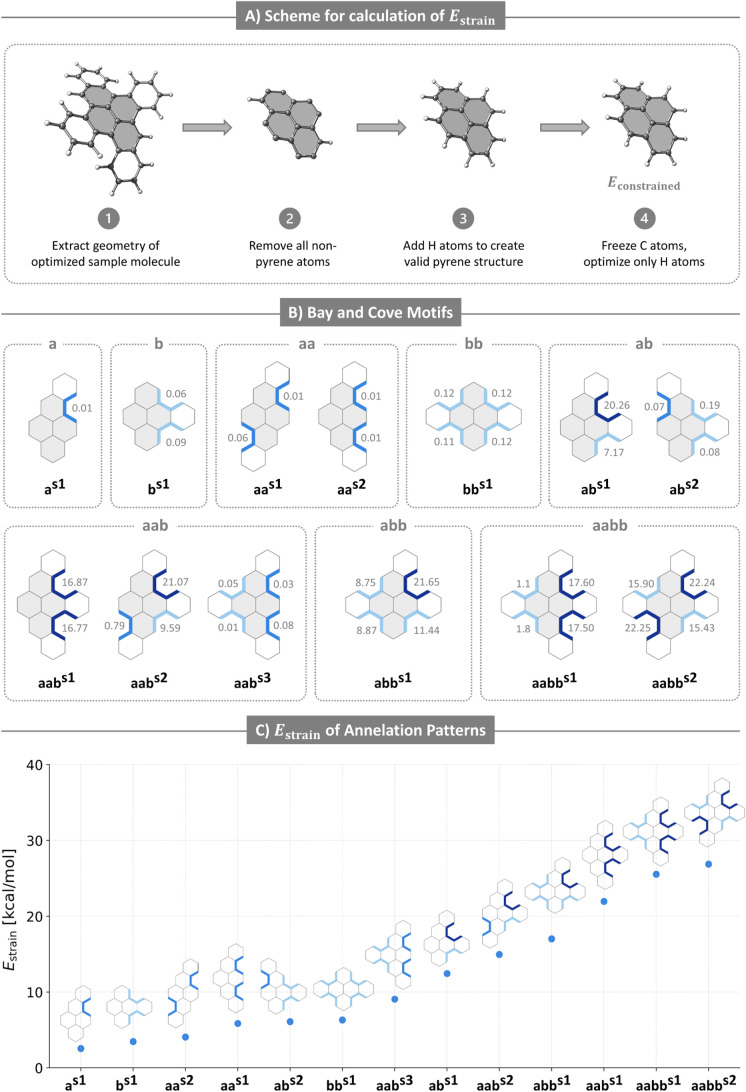
A) Scheme for calculation of *E*_constrained_. B) Sample molecules for estimating *E*_strain_. Dihedral angles are noted next to coves (dark blue), *a*-annelation bays (blue), and *b*-annelation bays (light blue) (for coves, the average of the two dihedral angles is given). C) Plot of *E*_strain_ for the sample molecules, ordered from lowest to highest. In all panels, double bonds are omitted for clarity.

The optimized geometry of each sample molecule was extracted from our data set. All nonpyrene atoms were removed and hydrogens were placed instead of the carbons directly annelated to the pyrene core, to form a chemically valid pyrene structure. A constrained optimization procedure was carried out, in which the carbon atoms of the pyrene core were kept frozen and only the hydrogens were allowed to relax. The resulting energy was termed *E*_constrained_ (Figure 7A). We defined *E*_strain_ = *E*_constrained_ – *E*_pyrene_ (the difference in energy between each constrained pyrene core and the fully relaxed native pyrene system), as a reasonable approximation of the strain incurred by the pyrene core for each annelation pattern.

#### 2.2.2 Correlation of *E*_strain_ with Structure

To analyze *E*_strain_ in terms of structural motifs, we counted the numbers of cove and bay motifs^45^ (highlighted in Figure 7B) for each pattern, distinguishing between bays stemming from *a*-annelation and *b*-annelation. Table 2 details the three metrics *n*_coves_, $n_{\mathrm{bays}}^{a}$ , and $n_{\mathrm{bays}}^{b}$ for each of the 13 sample molecules.

**Table 2:** *n*_coves_, $n_{\mathrm{bays}}^{a}$ , $n_{\mathrm{bays}}^{b}$ , and *n*_strain_ Values for Each of the 13 Structures Included in the Strain Analysis^a^

Sample Mol.	*n*_coves_	$n_{\mathrm{bays}}^{a}$	$n_{\mathrm{bays}}^{b}$	*n*_strain_ ^b^
**a** ^**s1**^	0	1	0	1.0
**b** ^**s1**^	0	0	2	1.4
**aa** ^**s1**^	0	2	0	2.0
**aa** ^**s2**^	0	2	0	2.0
**ab** ^**s2**^	0	1	2	2.4
**bb** ^**s1**^	0	0	4	2.8
**aab** ^**s3**^	0	2	2	3.4
**ab** ^**s1**^	1	0	1	5.0
**aab** ^**s2**^	1	1	1	6.0
**abb** ^**s1**^	1	0	3	6.4
**aab** ^**s1**^	2	0	0	8.6
**aabb** ^**s1**^	2	0	2	10.0
**aabb** ^**s2**^	2	0	2	10.0

a The molecules are arranged in order from lowest to highest *E*_strain_.

b $n_{\mathrm{strain}}=n_{\mathrm{bays}}^{a}+0.7\cdot n_{\mathrm{bays}}^{b}+4.3\cdot n_{\mathrm{coves}}$ .

Our expectation was that these nonplanar motifs incur geometric distortion and increase *E*_strain_. We further expected that coves are more destabilizing than bays, due to their greater distortion. Unsurprisingly, both Table 2 and Figure 7C (a scatter plot of *E*_strain_ for all test molecules) reveal a strong relationship between *n*_coves_ and *E*_strain_: molecules with *n*_coves_ = 0 have the lowest *E*_strain_, followed by molecules with *n*_coves_ = 1, and finally by molecules with *n*_coves_ = 2 (the maximal number possible in our test set). The patterns that contain subgroups (**ab** and **aab**) are particularly supportive of our hypothesis, as they show a clear increase in *E*_strain_ with an increase in *n*_coves_, while maintaining the same numbers and types of annelations.

The bay motifs also exhibit an effect, albeit a subtler one. This effect is obscured by the stronger cove effect and only becomes obvious when looking at *n*_coves_ = 0 cases. The molecules **a** ^**s1**^, **b** ^**s1**^, **aa** ^**s1**^, **aa** ^**s2**^, **bb** ^**s1**^, and **aab** ^**s1**^ are all higher in energy than the parent pyrene, despite having *n*_coves_ = 0, indicating that bay motifs cause minor but non-negligible geometric distortion, which is corroborated by the dihedral angles (detailed in Figure 7B). From the values for **a** ^**s1**^ and **b** ^**s1**^, it can be seen that single bay motifs cause a very minor distortion from planarity and that *a*-annelation causes less distortion than *b*-annelation. Also, a *b*-annelation creates two bays, whereas an *a*-annelation creates only one. Overall, these observations are consistent with the *E*_strain_ values for both cases: 2.6 and 3.5 kcal/mol, respectively, for **a** ^**s1**^ and **b** ^**s1**^. The origin of this distortion has been attributed to H–H steric repulsion, which varying methods have estimated to be in the range of 1.3–3.4 kcal/mol (in good agreement with our own results).^46–50^

We next attempted to find a quantitative relationship between the *n*_coves_, $n_{\mathrm{bays}}^{a}$ , and $n_{\mathrm{bays}}^{b}$ metrics and *E*_strain_. The obvious approaches—correlation between *E*_strain_ and *n*_coves_ or the sum of motifs—gave unsatisfying results (see Section S4 of the Supporting Information). The former, because it omits the non-negligible bay effects and therefore lacks sensitivity; the latter, because it assumes the contribution of each motif is identical, which is clearly not the case. Therefore, we designed a weighting scheme to quantify the relative strength of each motif. We selected three representative cases featuring only one type of motif: the *E*_strain_ of **a** ^**s1**^ accounts for $n_{\mathrm{bays}}^{a}=1$ ; the *E*_strain_ of **b** ^**s1**^ accounts for $n_{\mathrm{bays}}^{b}=2$ ; and the *E*_strain_ of **aab** ^**s1**^ accounts for *n*_coves_ = 2. The *E*_strain_ value for each systems was divided by the lowest value (that of **a** ^**s1**^, 2.6 kcal/mol) and by the number of motifs it accounts for, affording a relative weighting of 1:0.7:4.3 for *a*-bay:*b*-bay:cove, respectively. Based on this ratio, we defined for each molecule a single metric to encompass all strain effects, $n_{\mathrm{strain}}=n_{\mathrm{bays}}^{a}+0.7\cdot n_{\mathrm{bays}}^{b}+4.3\cdot n_{\mathrm{coves}}$ (Table 2, fourth column). Figure 8 presents a scatter plot of *n*_strain_ against the DFT-calculated *E*_strain_ for all 13 sample molecules. The remarkable linear correlation validates our approach to quantifying the strain and strongly substantiates the structure–property relationships we describe.

**Figure 8:**
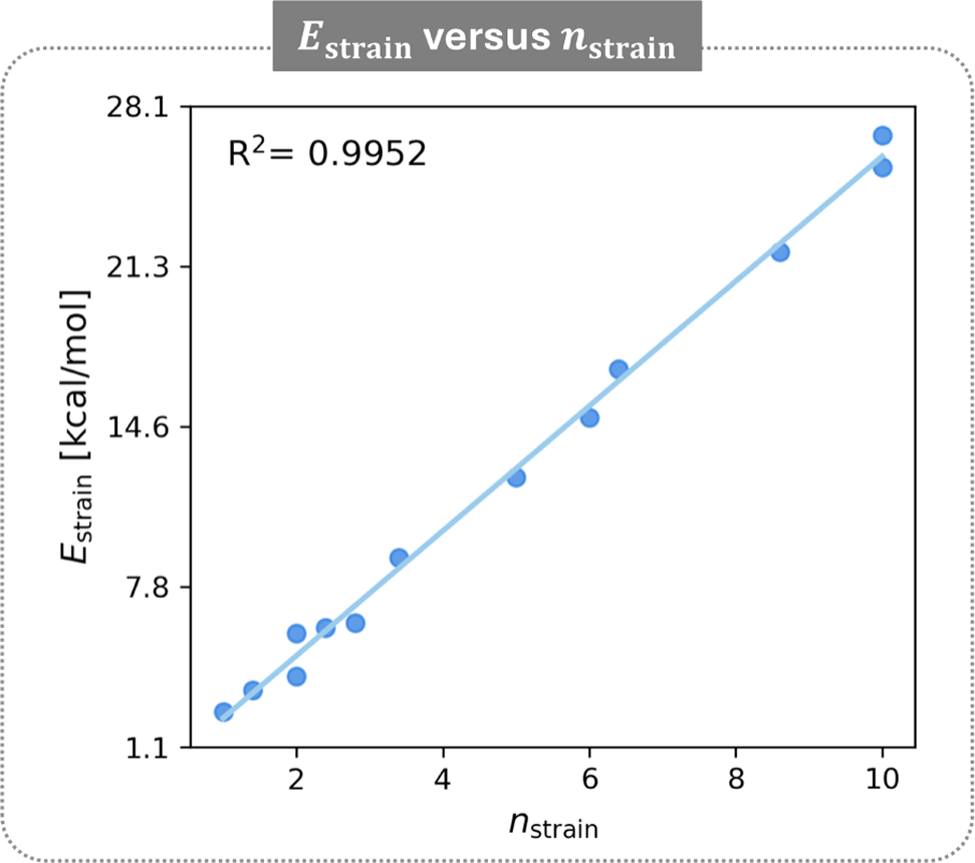
Scatter plot of *E*_strain_ versus *n*_strain_ for the sample molecules with regression line.

#### 2.2.3 Understanding *E*_rel_

We began this section with the hypothesis that the behavior of *E*_rel_ is anomalous to the other five properties because it contains contributions from both conjugation and strain. Having defined two new metrics—ΔClar for the conjugation and *n*_strain_ for the strain—we wanted to see if these could be combined to explain the observed order of patterns in *E*_rel_ seen in Figure 2.

To answer this question, we fit a linear equation of the type: $E_{rel}^{avg}=\alpha \cdot n_{strain}^{avg}+\beta \cdot \Delta \mathrm{Clar}+\gamma$ . We note that each annelation pattern is characterized by a single ΔClar value, but may have multiple *n*_strain_ values for the individual subgroups. Therefore, we used the average *n*_strain_ of the pattern. We then optimized the coefficients α, β and γ to produce the best fit between the predicted $E_{rel}^{avg}$ values and those from the data set (see Section S5 in the Supporting Information for further details on the fitting procedure). The best fit (*R* ^2^ = 0.8789) was obtained for the equation $E_{rel}^{avg}=0.8\cdot n_{strain}^{avg}-0.3\cdot \Delta \mathrm{Clar}+13.4$ (Figure 9). Furthermore, the equation very closely reproduces the order of patterns for *E*_rel_. The predicted $E_{rel}^{avg}$ values follow the order: **b** < **a** < **bb** < **aa** < **ab** < **abb** < **aab** < **aabb**. Only groups **b** and **bb** are not in their original locations, due to relatively small discrepancies—**b** is underestimated by ∼2 kcal/mol and **bb** is overestimated by ∼1 kcal/mol.

**Figure 9:**
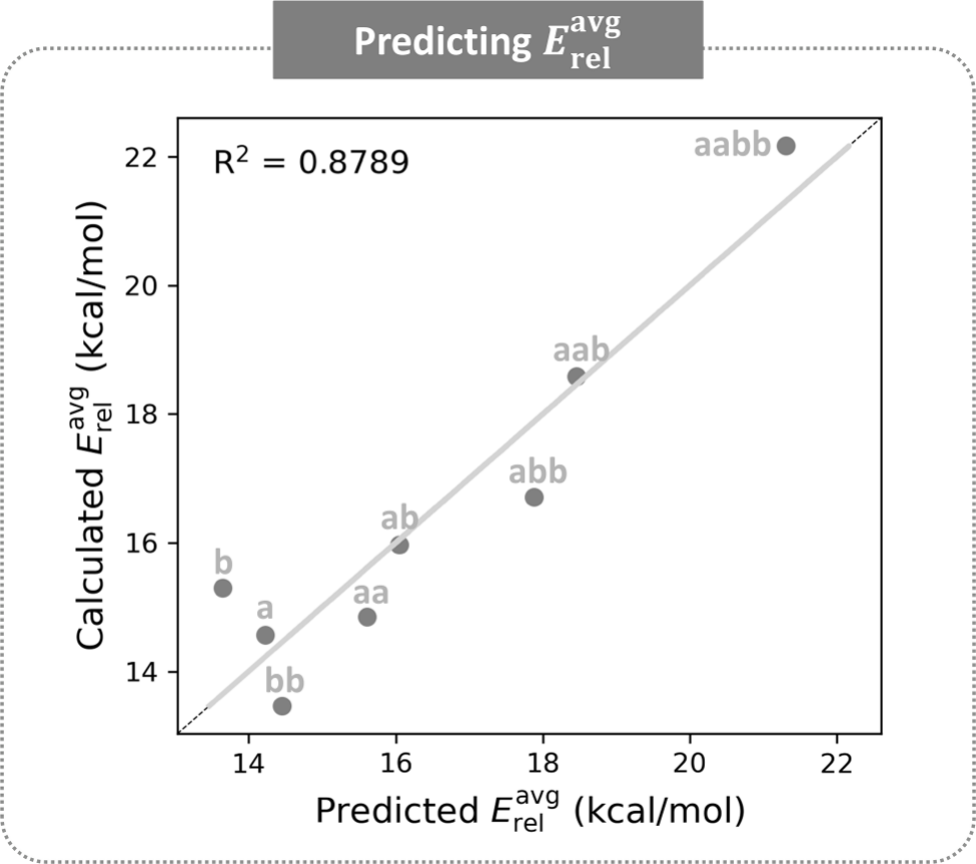
Scatter plot of $E_{rel}^{avg}$ values predicted with our metrics versus $E_{rel}^{avg}$ values obtained from the DFT-calculated data.

Considering the simplicity of our model versus the diversity of this chemical space, the agreement is quite gratifying, and it indicates that our approach largely manages to capture the important structural features determining the relative stability of pyrene-based PBHs. Indeed, we note that our approach is in excellent alignment with the recent publication of Gregory and Karton, in which dihedral angle deviations are used to predict the thermodynamic stability of *peri*-condensed PBHs (published while the present manuscript was under review).^100^ Our analysis sheds further light on the factors determining the stability of pyrene-based PBHs. The fitting equation shows that both the strain and the conjugation are important, while the size and sign of α and β highlight their individual contributions. Not surprisingly, α > 0 while β < 0, signifying the destabilization caused by strain versus the stabilization afforded by delocalization/aromaticity. In addition, α > β, indicating that strain plays the dominant role. In terms of accuracy, of course, further modifications to the model could improve the agreement; however, the main advantages of our approach are its interpretability and the fact that all necessary features can be found with a back-of-the-envelope calculation. Moreover, in this case our main goal was to validate our conceptual framework, and not to provide a highly accurate surrogate model for DFT calculations.

## 3 Conclusions

In this work, we present a comprehensive and systematic study of 4,766 pyrene-based PBHs, elucidating how annelation patterns modulate electronic properties through additive and opposing effects. By classifying these molecules according to *a*-annelation and *b*-annelation, we uncovered consistent structure–property relationships across key π-system properties: HOMO and LUMO energies, Δ*E*_H–L_, aIP, and aEA. Our classification reveals that *a*-annelation raises the HOMO and lowers the LUMO, while *b*-annelation has the inverse effect; we further find that these influences combine in an additive fashion, enabling fine-tuned control of molecular properties.

The Clar resonance structure analysis provides a compelling rationalization for these observations. We show that the difference between fixed and migrating Clar sextets (ΔClar) serves as a powerful predictor of electronic behavior, with fixed sextets correlating to larger Δ*E*_H–L_ values and migrating sextets counteracting this. This metric successfully captures the trends across annelation patterns, especially when considering molecules of equal size.

Further, we explore the interplay between local pyrene annelation patterns and extended conjugation motifs. Our results demonstrate that linear stretches of benzenoid annelation (quantified by *n*_LL_) can dominate electronic properties, effectively superseding local pyrene effects in sufficiently extended systems. Importantly, the susceptibility of the molecular properties to linear extension is found to depend on the specific annelation pattern, offering additional design flexibility.

The thermodynamic stability of the pyrene isomers (*E*_rel_) is shown to be strongly influenced by geometric distortion and therefore deviates from the other property trends. Through constrained geometry optimizations, we develop a quantitative strain metric (*E*_strain_) and identify a weighted motif-based model incorporating bay and cove contributions. This model, when combined with ΔClar, provides a remarkably intuitive framework for understanding *E*_rel_.

Altogether, this study offers a unified conceptual framework that integrates annelation geometry, extended conjugation, and geometric distortion. Thanks to this classification, we demonstrate that, while annelation patterns capture average behavior, other structural factors (e.g., molecular size, conjugation length, strain) are essential for finer-grained prediction. These insights pave the way for rational design of functional PBHs based on pyrene, enabling precise tailoring of molecular properties for applications in organic electronics, (opto)­electronics, and beyond.

## 4 Computational Methods

All data used in this study were taken from the COMPAS-3D data set.^8^ COMPAS-3D contains all possible pc-PBH isomers consisting of 4 ≤ *n*_rings_ ≤ 10. Molecular geometries were optimized at the CAM-B3LYP^51–55^ ‑D3BJ^56^/def2-SVP^57,58^ level and the electronic properties were calculated at the CAM-B3LYP-D3BJ/aug-cc-pVDZ^59–61^ level. For further information on the choice of functional/basis set combination, see Section S1 of the Supporting Information. For this study, we extracted all 4,766 isomers containing a single pyrene moiety (pyrene itself is not included in this data set, thus the data in this study are in the range of 5 ≤ *n*_rings_ ≤ 10). All molecules in this study were determined to be minima on their respective potential energy surfaces (i.e., *N*_imag_ = 0) and to have a closed-shell ground state.^62^

## Supplementary Material



## Data Availability

The data underlying this study are available in the published article, in its Supporting Information, and openly available in GitLab at https://gitlab.com/porannegroup/compas.

## Notes

Additional details on choice of computational level of theory and benchmarking, additional details on the classification method and the data contained in each subgroup, additional discussion as mentioned in the text, input templates, additional details on linear regression model fitting (PDF)

## References

[1] Anthony J. E. (2006). Functionalized
Acenes and Heteroacenes for Organic Electronics. Chem. Rev..

[2] Lee C. W., Kim O. Y., Lee J. Y. (2014). Organic
materials for organic electronic
devices. J. Ind. And Eng. Chem..

[3] Tsuji H., Nakamura E. (2017). Design and Functions
of Semiconducting Fused Polycyclic
Furans for Optoelectronic Applications. Acc.
Chem. Res..

[4] Aumaitre C., Morin J.-F. (2019). Polycyclic Aromatic Hydrocarbons as Potential Building
Blocks for Organic Solar Cells. Chem. Rec..

[5] Chen C., Zhang Y., Wang X.-Y., Wang J.-Y., Pei J. (2023). Boron- and
Nitrogen-Embedded Polycyclic Arenes as an Emerging Class of Organic
Semiconductors. Chem. Mater..

[6] Wahab A., Pfuderer L., Paenurk E., Gershoni-Poranne R. (2022). The COMPAS
Project: A Computational Database of Polycyclic Aromatic Systems.
Phase 1: cata-Condensed Polybenzenoid Hydrocarbons. J. Chem. Inf. Model..

[7] Mayo
Yanes E., Chakraborty S., Gershoni-Poranne R. (2024). COMPAS-2:
a dataset of cata-condensed hetero-polycyclic aromatic systems. Sci. Data.

[8] Wahab A., Gershoni-Poranne R. (2024). COMPAS-3:
a dataset of peri-condensed polybenzenoid
hydrocarbons. Phys. Chem. Chem. Phys..

[9] Chakraborty S., Almog I., Gershoni-Poranne R. (2025). COMPAS-4:
A Data Set of (BN)­1 Substituted
Cata-Condensed Polybenzenoid Hydrocarbons–Data Analysis and
Feature Engineering. J. Chem. Inf. And Model.

[10] Markert G., Paenurk E., Gershoni-Poranne R. (2021). Prediction
of Spin Density, Baird-Antiaromaticity,
and Singlet-Triplet Energy Gap in Triplet-State Polybenzenoid Systems
from Simple Structural Motifs. Chem.-Eur. J..

[11] Fite S., Wahab A., Paenurk E., Gross Z., Gershoni-Poranne R. (2023). Text-based
representations with interpretable machine learning reveal structure-property
relationships of polybenzenoid hydrocarbons. J. Phys. Org. Chem..

[12] Weiss T., Wahab A., Bronstein A. M., Gershoni-Poranne R. (2023). Interpretable
Deep-Learning Unveils Structure-Property Relationships in Polybenzenoid
Hydrocarbons. J. Org. Chem.

[13] Weiss T., Mayo Yanes E., Chakraborty S., Cosmo L., Bronstein A. M., Gershoni-Poranne R. (2023). Guided diffusion for inverse molecular design. Nat. Comp. Sci..

[14] Reeta
Felscia U., Rajkumar B. J. M., Briget Mary M. (2018). Charge transport
properties of pyrene and its derivatives: optoelectronic and nonlinear
optical applications. J. Mater. Sci.

[15] Song X., Wang X., Liu W., Chen X., Li S., Islam M. S., Li L., Zhao X., Redshaw C., Zhao Y., Cao C. C., Feng X. (2025). Organic Photo-Responsive
Piezoelectric Materials Based on Pyrene Molecules for Flexible Sensors. Adv. Electron. Mater..

[16] Walter M., Kravchyk K. V., Böfer C., Widmer R., Kovalenko M. V. (2018). Polypyrenes
as High-Performance Cathode Materials for Aluminum Batteries. Adv. Mater..

[17] Cho H., Lee S., Cho N. S., Jabbour G. E., Kwak J., Hwang D.-H., Lee C. (2013). High-Mobility Pyrene-Based Semiconductor for Organic Thin-Film Transistors. ACS Appl. Mater. Interfaces.

[18] Usman K., Islam A., Ullah Shah S. H., Javaid K., Amin A., Mustafa Z., Wattoo A. G., Abbas N., Ge Z. (2021). Fluorescent
pyrene-imidazole material for deep-blue organic light-emitting devices. Opt. Mater..

[19] Oh H.-Y., Lee C., Lee S. (2009). Efficient blue organic light-emitting diodes using
newly-developed pyrene-based electron transport materials. Org. Electron..

[20] Feng X., Wang X., Redshaw C., Tang B. Z. (2023). Aggregation
behaviour
of pyrene-based luminescent materials, from molecular design and optical
properties to application. Chem. Soc. Rev..

[21] Barry, N. P. E. ; Therrien, B. ; Sadjadi, S. Organic Nanoreactors, Academic Press: Boston, 2016; pp. 421-461. 10.1016/B978-0-12-801713-5.00013-6.

[22] Ayyavoo K., Velusamy P. (2021). Pyrene based materials
as fluorescent probes in chemical
and biological fields. New J. Chem..

[23] Kowser Z., Rayhan U., Akther T., Redshaw C., Yamato T. (2021). A brief review
on novel pyrene based fluorometric and colorimetric chemosensors for
the detection of Cu 2+. Mater. Chem. Front..

[24] Prabakaran G., David C. I., Nandhakumar R. (2023). A review on
pyrene based chemosensors
for the specific detection on d-transition metal ions and their various
applications. J. Environ. Chem. Eng..

[25] Shellaiah M., Sun K.-W. (2022). Pyrene-Based AIE
Active Materials for Bioimaging and
Theranostics Applications. Biosensors.

[26] Wang X.-Q., Ling Q.-H., Wang W., Xu L. (2020). Pyrene-based metallocycles
and metallocages: more than fluorophores. Mater.
Chem. Front..

[27] Yang Y., Peng S., Chen S., Kang F., Fan J., Zhang H., Yu X., Li J., Zhang Q. (2024). Pyrene-based
covalent organic frameworks (PyCOFs): a review. Nanoscale Horiz..

[28] Pelin
Kinik F., Ortega-Guerrero A., Ongari D., Ireland C. P., Smit B. (2021). Pyrene-based metal organic frameworks: from synthesis to applications. Chem. Soc. Rev..

[29] Dong X., Zhao H., Zhang K., Lang X. (2024). Pyrene-based porous
organic materials for visible light photocatalysis. Coordin Chem. Rev..

[30] Guo D., Li H., Xu Z., Nie Y. (2023). Development of pyrene-based MOFs
probe for water content and investigations on their mechanochromism
and acidochromism. J. Alloy. Compd..

[31] Keller S. N., Veltri N. L., Sutherland T. C. (2013). Tuning
Light Absorption in Pyrene:
Synthesis and Substitution Effects of Regioisomeric Donor-Acceptor
Chromophores. Org. Lett..

[32] Bédard A.-C., Vlassova A., Hernandez-Perez A. C., Bessette A., Hanan G. S., Heuft M. A., Collins S. K. (2013). Synthesis,
Crystal Structure and
Photophysical Properties of Pyrene-Helicene Hybrids. Chem.-Eur. J..

[33] Shi Y.-R., Wei H.-L., Shi Y.-T., Liu Y.-F. (2017). Theoretical study
of pyrene derivatives as high performance organic semiconductor materials. Synth. Met..

[34] Feng X., Hu J.-Y., Redshaw C., Yamato T. (2016). Functionalization of
Pyrene To Prepare Luminescent Materials–Typical Examples of
Synthetic Methodology. Chem.-Eur. J..

[35] Radenković S., Tošović J., Havenith R. W. A., Bultinck P. (2015). Ring currents
in benzo- and benzocyclobutadieno-annelated biphenylene derivatives. ChemPhyschem.

[36] Radenković S., Đorđević S., Nikolendžić M. (2024). Effect of
Benzo-Annelation on Triplet State Energies in Polycyclic Conjugated
Hydrocarbons. Chem.-Eur. J..

[37] Radenković S., Đorđević S. (2023). Effect of benzo-annelation on magnetically
induced current density. Chem. Phys. Lett..

[38] Clar, E. The Aromatic sextet; Wiley-Interscience, 1972.

[39] Rondia, D. ; Cooke, M ; Haroz, R. K. Mobile source emissions including policyclic organic species; Springer, 1983; pp. 49–58.

[40] Balaban A. T. (2004). Clar Formulas:
How to Draw and How not to Draw Formulas of Polycyclic Aromatic Hydrocarbons. Polycyclic Aromat. Compd..

[41] Rieger R., Müllen K. (2010). Forever young: polycyclic aromatic
hydrocarbons as
model cases for structural and optical studies. J. Phys. Org. Chem..

[42] Solà M. (2013). Forty years
of Clar’s aromatic Π-sextet rule. Front. Chem..

[43] Randić M. (2014). Novel insight
into Clar’s aromatic Π-sextets. Chem. Phys. Lett..

[44] Gershoni-Poranne R., Gibson C. M., Fowler P. W., Stanger A. (2013). Concurrence
between
Current Density, Nucleus-Independent Chemical Shifts, and Aromatic
Stabilization Energy: The Case of Isomeric 4 - and 5 Phenylenes. J. Org. Chem..

[45] Gutman, I. ; Cyvin, S. J. Introduction to the Theory of Benzenoid Hydrocarbons; Springer: Berlin, Heidelberg, 1989. 10.1007/978-3-642-87143-6.

[46] Nagano Y., Nakano M. (2003). Strain energy of phenanthrene. J. Chem. Thermodyn..

[47] Poater J., Bickelhaupt F. M., Solà M. (2007). Didehydrophenanthrenes:
Structure,
Singlet-Triplet Splitting, and Aromaticity. J. Phys. Chem. A.

[48] Vashchenko A. V., Borodina T. N. (2013). H-H interaction in phenanthrene: Attraction or repulsion?. J. Struct. Chem..

[49] Radenković S., Gutman I., Đorđević S. (2015). Strain in
strain-free benzenoid hydrocarbons: The case of phenanthrene. Chem. Phys. Lett..

[50] Poater J., Duran M., Solà M. (2018). Aromaticity
Determines the Relative
Stability of Kinked vs. Straight Topologies in Polycyclic Aromatic
Hydrocarbons. Front. Chem..

[100] Gregory K. P., Karton A. (2025). Big-Data Analysis of
Geometric Descriptors
as Efficient Predictors of Energetic Stability in Nonplanar Polycyclic
Aromatic Hydrocarbons. J. Comput. Chem..

[51] Becke A. D. (1993). Density-functional
Thermochemistry III. The Role of Exact Exchange. J. Chem. Phys..

[52] Lee C., Yang W., Parr R. G. (1988). Development of the Colle-Salvetti
Correlation-energy Formula Into a Functional of the Electron Density. Phys. Rev. B.

[53] Miehlich B., Savin A., Stoll H., Preuss H. (1989). Results Obtained with
the Correlation Energy Density Functionals of Becke and Lee, Yang
and Parr. Chem. Phys. Lett..

[54] Hertwig R. H., Koch W. (1997). On the Parameterization
of the Local Correlation Functional. What
is Becke-3-LYP?. Chem. Phys. Lett..

[55] Yanai T., Tew D. P., Handy N. C. (2004). A new hybrid
exchange-correlation
functional using the Coulomb-attenuating method (CAM-B3LYP). Chem. Phys. Lett..

[56] Grimme S., Antony J., Ehrlich S., Krieg H. (2010). A Consistent
and Accurate *ab initio* Parametrization of Density
Functional Dispersion
Correction (DFT-D) for the 94 Elements H-Pu. J. Chem. Phys..

[57] Johnson E.
R., Becke A. D. (2005). A post-Hartree-Fock
Model of Intermolecular Interactions. J. Chem.
Phys..

[58] Grimme S., Ehrlich S., Goerigk L. (2011). Effect of
the Damping Function in
Dispersion Corrected Density Functional Theory. J. Comput. Chem..

[59] Dunning T. H. (1989). Gaussian
basis sets for use in correlated molecular calculations. I. The atoms
boron through neon and hydrogen. J. Chem. Phys..

[60] Kendall R. A., Dunning T. H., Harrison R. J. (1992). Electron
affinities of the first-row
atoms revisited. Systematic basis sets and wave functions. J. Chem. Phys..

[61] Woon D. E., Dunning T. H. (1993). Gaussian basis sets for use in correlated molecular
calculations. III. The atoms aluminum through argon. J. Chem. Phys..

[62] Wahab A., Gershoni-Poranne R. (2025). Accelerated diradical character assessment in large
datasets of polybenzenoid hydrocarbons using xTB fractional occupation. Phys. Chem. Chem. Phys..

