# Supplementary material for: From Rings to Properties: Understanding the Effect of Annelation on Pyrene
Source: J Org Chem. 2025 Aug 29;90(36):12667–77. doi: 10.1021/acs.joc.5c01401 (PMC12442067; doi:10.1021/acs.joc.5c01401)
Supplement: Supplementary file 1 [file jo5c01401_si_001.pdf]

Supporting Information

From Rings to Properties: Understanding the Effect of Annelation on Pyrene

Alexandra Wahab[a] and Renana Gershoni-Poranne[a,b]

[a] *The Laboratory for Organic Chemistry, Department of Chemistry and Applied Biosciences, ETH Zürich, 8093 Zürich, Switzerland*
[b] *The Schulich Faculty of Chemistry and the Resnick Sustainability Center for Catalysis, Technion – Israel Institute of Technology, Haifa 32000, Israel*

e-mail: rporanne@technion.ac.il

Contents

S1 Computational Details **S3**
S1.1 Choice of Level of Theory . . . S3
S1.1.1 Benchmarking . . . S3
S1.2 Input template for full optimizations . . . S9
S1.3 Input template for constrained optimizations . . . S10

S2 Additional Information on the Data Classification **S12**
S2.1 Annelation Pattern Classification . . . S12
S2.2 Data Overview . . . S13

S3 Size Dependency of Properties and Defined Features **S15**
S3.1 Molecular Properties . . . S15
S3.2 Defined Features . . . S17

S4 Additional ΔClar **Analysis** **S18**

S5 Correlation of E_{strain} **with Structure** **S20**

S6 Feature optimization to predict $E_{\mathrm{rel}}^{\mathrm{avg}}$ **S21**

S1 Computational Details

All geometries and properties of fully-optimized molecules used in this study were taken from the COMPAS-3D dataset. Although these calculations were not carried out in the course of this work, we provide all necessary computational details for reproduction of the work, including templates of the input files used.

For the analysis of strain energy, we also generated in the course of this work constrained-optimization geometries, as detailed in the main text. The input templates for these calculations are also provided herein.

S1.1 Choice of Level of Theory

The COMPAS-3D dataset was calculated with ORCA version 5.0.3.[S1,S2] We used the CAM-B3LYP[S3–S7] functional and the def2-SVP[S8] basis set for optimization, followed by a single point with a larger basis set (aug-cc-pVDZ)[S9–S11] with Grimme's D3[S12] dispersion correction and the Becke-Johnson damping scheme.[S13,S14]

These methods were selected following a literature search[S15] and a subsequent benchmarking procedure (see below).

S1.1.1 Benchmarking

The following text has been reproduced with permission from Section S2 of the SI of Reference S16 (copyright 2024, the Royal Society of Chemistry). It details the benchmarking procedure undertaken to choose the functional and basis sets used in the generation of the COMPAS-3D dataset, from which the data for the current investigation were extracted.

We performed a benchmarking procedure to identify a cost-effective and accurate functional/basis set combination for TD-DFT calculations of the T_1 and S_1 excited states. Although COMPAS-3 contains only closed-shell ground-state molecules by design, future installments of the COMPAS Project are focused on excited states. To ensure compatibility for future analyses comparing the ground and excited states, we needed to choose a level of theory that would be suitable for both cases. Since excited-state calculations are much more sensitive to method choice, we benchmarked the methods for excited states to calculate COMPAS-3D. The selected methods have been extensively used for ground-state PASs and are generally considered appropriate for them.

We selected CAM-B3LYP[S7] as our functional as it was found to be one of the best performing functionals in a recent benchamrking study conducted by Head-Gordon and co-workers.[S15] The choice of which basis set to test was also based on the benchmarking paper, where the authors compared multiple basis set families – aug-cc-pVXZ ($X = D, T, Q$),[S9–S11] d-aug-cc-pVXZ,[S17] and the def2 series[S8,S18] – on a select few functionals. From their results, we selected the best performing and less resource consuming basis set family—aug-cc-pVXZ. We compared the performances of aug-cc-pVDZ (aDZ) and aug-cc-pVTZ (aTZ) on the same benchmarking set previously use in our dft benchmarking for compas-1 (see Figure S1).

aTZ is significantly more costly than aDZ, especially as the calculated system gets larger. Nonetheless, it has the advantage of having a dedicated auxiliary basis set implemented in ORCA 5.0.3, which is not the case for aDZ. Auxiliary basis sets are important for the simultaneous fitting of Coulomb and exchange integrals. In other words, it is essential for reducing calculation run times. Thus, we tested three solutions to remedy this problem:

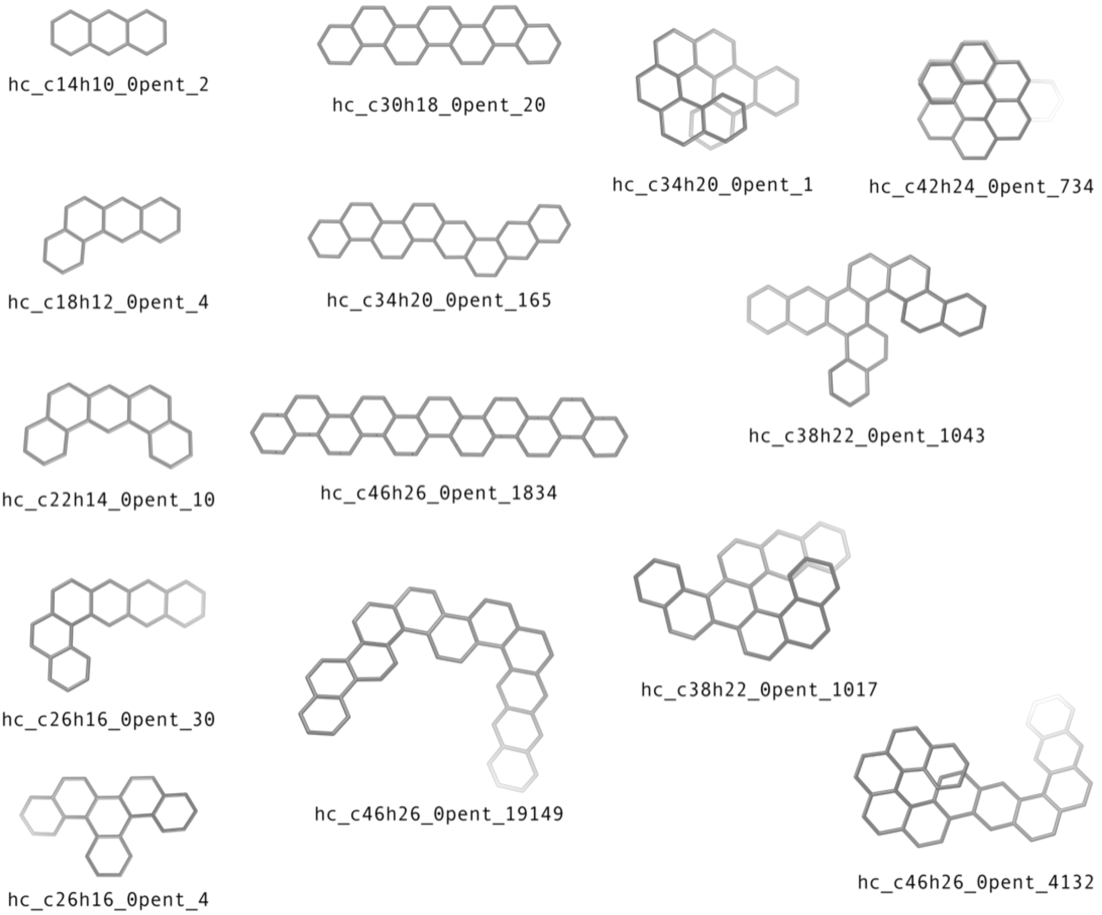

Figure S1. Sample of 14 molecules from COMPAS-1 with 3 to 11 rings used for benchmarking. Reproduced with permission from reference S16. Copyright 2024 Royal Society of Chemistry.

(1) using the def2/J basis set (test 1); (2) using a pcd with 'AutoAux', meaning that the adequate auxiliary basis set is chosen automatically and the pcd will deal with linear dependencies that may arise (test 2); (3) using aug-cc-pVTZ/JK auxiliary basis set (test 3). We also added a fourth option (final), which speeds up the calculations significantly (see Figure S2). All five set-ups are described in Table S1.

Some of the calculations were harder to converge, notably 8 out of the 14 molecules required adjustment when calculated with the aTZ basis set. We gradually increased 'DIISMaxEq' value until convergence. This keyword indicates the number of Fock matrices to be remembered for the DIIS extrapolation. The default value is 5, but difficult systems might require a value between 15-40. If convergence was not reached, we lowered the 'DirectResetFreq' value to 5, then to 1. This keyword controls how often the full Fock matrix is rebuilt. The default value is 15. This keyword greatly affects the run time of the computations as building the Fock matrix is a costly computation. The molecules that required these changes are show in Table S2.

To check that the more cost effective choices would not lead to large deviations of the calculated properties, we compared the vertical T_1 (Table S3) and vertical S_1 (Table S4) excitation energies to aTZ, which should be the most accurate from the set-ups compared. All set-ups showed extremely good correlations with the aTZ energies with slopes equal to 1 and intercepts going through the origin, and R^2 varying from 0.9999 to 1.0000.

The set-up we converged on (referred to as final in the tables and plots) showed good

Table S1. The different set-ups compared for the benchmarking.

	**Set up details**
**aTZ**	CAM-B3LYP functional / aug-cc-pVTZ basis set / aug-cc-pVTZ/JK auxiliary
**Test 1**	CAM-B3LYP functional / aug-cc-pVDZ basis set / Def2/JK auxiliary
**Test 2**	CAM-B3LYP functional / aug-cc-pVDZ basis set<br>`%basis`<br>`auxj "AutoAux"`<br>`PCDTrimAuxJ  Coulomb  # Trim the AuxJ basis in the`<br>`                      # Coulomb metric`<br>`PCDThresh -1          # Threshold for the PCD:`<br>`                      # chosen automatically if >0`<br>`end`
**Test 3**	CAM-B3LYP functional with aug-cc-pVDZ basis set and aug-cc-pVTZ/JK auxiliary
**Final**	CAM-B3LYP functional with aug-cc-pVDZ basis set<br>`%basis`<br>`newgto C "aug-cc-pVDZ" end  # set aug-cc-pVDZ as the`<br>`                            # basis set for C atoms`<br>`auxj "aug-cc-pVTZ/JK"       # auxiliary basis set`<br>`end`

Table S2. Changes made to the %scf block.

**ID**	**Molecule**	**aTZ**	**Test 1**	**Test 2**	**Test 3**	**Final**
**1**	hc_c14h10_0pent_2	-	-	-	-	-
**2**	hc_c18h12_0pent_4	-	-	DirectResetFreq 5	-	-
**3**	hc_c22h14_0pent_10	DIISMaxEq 20	-	-	-	-
**4**	hc_c26h16_0pent_30	-	-	-	-	-
**5**	hc_c26h16_0pent_4	-	-	-	-	-
**6**	hc_c30h18_0pent_20	DIISMaxEq 20	-	-	-	-
**7**	hc_c34h20_0pent_1	-	-	-	-	-
**8**	hc_c34h20_0pent_165	-	-	DirectResetFreq 1	-	-
**9**	hc_c38h22_0pent_1017	DIISMaxEq 20	-	-	-	-
**10**	hc_c38h22_0pent_1043	DIISMaxEq 15	-	-	-	-
**11**	hc_c42h24_0pent_734	DirectResetFreq 1	-	-	-	-
**12**	hc_c46h26_0pent_1834	DIISMaxEq 20	-	-	-	-
**13**	hc_c46h26_0pent_19149	DirectResetFreq 1	-	-	-	-
**14**	hc_c46h26_0pent_4132	DIISMaxEq 15	-	-	-	-

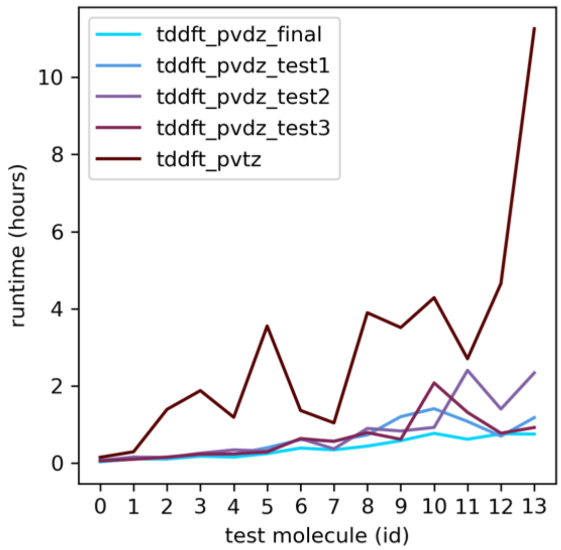

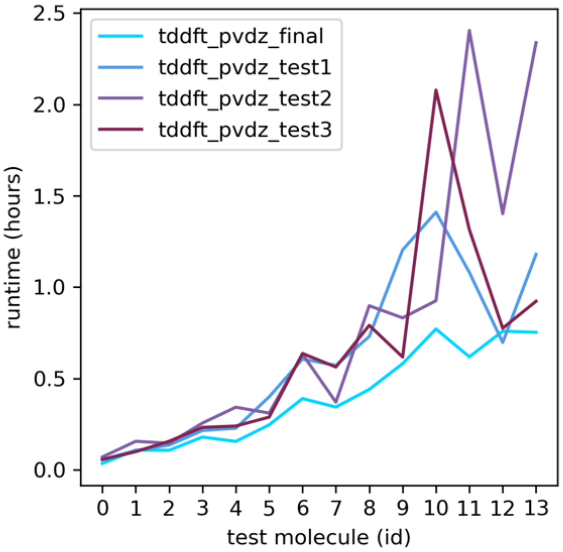

Figure S2. Total run times in hours obtained from jobs performed with 12 processors each. On the left: the five set-ups we compared. On the right: removed aTZ for better comparison of faster set-ups. Reproduced with permission from reference S16. Copyright 2024 Royal Society of Chemistry.

accuracy when compared to aTZ, while displaying the shortest run times (Figure S2). Additionally, the initial input did not require any modifications for all 14 test molecules and converged without issues, which is an important quality for high-throughput calculations that we want to perform.

Table S3. T_1 excitation energies for the five set-up that were tested. All values reported are in eV.

ID	Molecule	aDZ				aTZ
		**Final**	**Test 1**	**Test 2**	**Test 3**	
**1**	hc_c14h10_0pent_2	2.128	2.129	2.128	2.128	2.127
**2**	hc_c18h12_0pent_4	2.384	2.384	2.384	2.384	2.382
**3**	hc_c22h14_0pent_10	2.605	2.605	2.604	2.604	2.603
**4**	hc_c26h16_0pent_30	1.613	1.614	1.614	1.614	1.612
**5**	hc_c26h16_0pent_4	2.625	2.626	2.626	2.626	2.624
**6**	hc_c30h18_0pent_20	2.748	2.749	2.749	2.749	2.748
**7**	hc_c34h20_0pent_1	2.538	2.539	2.539	2.539	2.536
**8**	hc_c34h20_0pent_165	2.370	2.371	2.370	2.370	2.369
**9**	hc_c38h22_0pent_1017	2.113	2.114	2.114	2.114	2.112
**10**	hc_c38h22_0pent_1043	2.208	2.209	2.208	2.208	2.206
**11**	hc_c42h24_0pent_734	2.190	2.191	2.191	2.191	2.189
**12**	hc_c46h26_0pent_1834	2.697	2.698	2.697	2.697	2.697
**13**	hc_c46h26_0pent_19149	1.644	1.644	1.644	1.644	1.643
**14**	hc_c46h26_0pent_4132	2.038	2.039	2.039	2.039	2.037

Table S4. S_1 excitation energies for the five set-ups that were tested. All values are reported in eV.

ID	Molecule	aDZ				aTZ
		Final	Test 1	Test 2	Test 3	
**1**	hc_c14h10_0pent_2	3.726	3.726	3.725	3.725	3.715
**2**	hc_c18h12_0pent_4	3.825	3.824	3.824	3.824	3.816
**3**	hc_c22h14_0pent_10	3.749	3.748	3.749	3.749	3.744
**4**	hc_c26h16_0pent_30	2.981	2.981	2.981	2.981	2.972
**5**	hc_c26h16_0pent_4	3.653	3.653	3.653	3.653	3.648
**6**	hc_c30h18_0pent_20	3.771	3.771	3.771	3.771	3.766
**7**	hc_c34h20_0pent_1	3.431	3.431	3.431	3.431	3.426
**8**	hc_c34h20_0pent_165	3.494	3.493	3.493	3.493	3.489
**9**	hc_c38h22_0pent_1017	3.310	3.310	3.310	3.309	3.304
**10**	hc_c38h22_0pent_1043	3.410	3.410	3.410	3.410	3.401
**11**	hc_c42h24_0pent_734	3.230	3.230	3.230	3.230	3.225
**12**	hc_c46h26_0pent_1834	3.675	3.675	3.675	3.675	3.670
**13**	hc_c46h26_0pent_19149	2.999	2.999	2.998	2.998	2.990
**14**	hc_c46h26_0pent_4132	3.278	3.278	3.278	3.278	3.270

S1.2 Input template for full optimizations

The following input templates were used to optimize the geometries of the molecules studied in this work. In the template below, `CHARGE` is a placeholder for the charge (neutral, 0) of the molecule and `MULTIPLICITY` is a placeholder for the multiplicity (singlet, 1). The starting *xyz* coordinates used for optimization are xTB-optimized coordinates. The single point uses the DFT-optimized coordinates to calculated the properties with a larger basis set.

```
#### Optimization ####

# functional and basis set
! cam-b3lyp def2-svp

# accuracy, approximations, and dispersion corrections
! tightscf rijcosX def2/j d3bj

# type of calculation
! opt

# output control
! miniprint

%base"cam-b3lyp_def2-svp_opt_0"

# type of input; charge; multiplicity; input
*xyzfile CHARGE MULTIPLICITY xtbopt.xyz

$new_job
#### Single Point ####

# functional and basis set
! cam-b3lyp cc-pVDZ

# accuracy, approximation, and dispersion corrections
! tightscf rijcosx d3bj

# type of calculation
! sp PModel NoTrah

# output control
! normalprint printgap

# basis set block
%basis
newgto C "aug-cc-pVDZ" end   # set aug-cc-pVDZ as the basis set for C atoms
auxj "aug-cc-pVTZ/JK"        # auxiliary basis set
end

# scf block
%scf
sthresh 1e-6                 # set threshold for smallest eigen value
```

```
end

%base"cam-b3lyp_aug-cc-pvdz_sp_0"

# type of input; charge; multiplicity; input
*xyzfile CHARGE MULTIPLICITY cam-b3lyp_def2-svp_opt_0.xyz
```

S1.3 Input template for constrained optimizations

Constrained optimizations were performed as follows: the fully-optimized geometries of the thirteen selected molecules (detailed in the main text) were extracted from the COMPAS-3 dataset. For each of the molecules, all annelated rings were removed and any carbons directly bound to the pyrene core were replaced with hydrogens. The modified structures were subjected to an optimization procedure, in which the carbons of the pyrene core (atoms 1 to 16 corresponding to indices 0 to 15 in ORCA) were kept frozen and only the hydrogens were allowed to relax.

The level of theory used for the optimization was identical to the one used for the full optimizations. Optimizations were followed by single point calculations using the cc-PVDZ basis set, similarly to the COMPAS-3 dataset.

```

#### Optimization ####

# functional and basis set
! cam-b3lyp def2-svp

# accuracy, approximations, and dispersion corrections
! tightscf rijcosX def2/j d3bj

# type of calculation
! opt

# output control
! miniprint

# scf block
%scf
sthresh 1e-6   # set threshold for smallest eigen value
end

%geom
   Constraints
    {A * * * C}     # constrain all angles
    {D * * * * C}   # constrain all dihedral angles
    {C 0:15 C}      # constrain all cartesian coordinates of list of atoms
   end
end

%base"constrained_opt"

# type of input; charge; multiplicity; input
```

```
*xyzfile CHARGE MULTIPLICITY modified.xyz

$new_job
#### Single Point ####
#
# functional and basis set
! cam-b3lyp cc-pVDZ

# accuracy, approximation, and dispersion corrections
! tightscf rijcosx d3bj

# type of calculation
! sp PModel NoTrah

# output control
! normalprint printgap

# basis set block
%basis
newgto C "aug-cc-pVDZ" end   # set aug-cc-pVDZ as the basis set for C atoms
auxj "aug-cc-pVTZ/JK"        # auxiliary basis set
end

# scf block
%scf
sthresh 1e-6                 # set threshold for smallest eigen value
end

%base"constrained_opt_sp"

# type of input; charge; multiplicity; input
*xyzfile CHARGE MULTIPLICITY constrained_opt.xyz
```

S2 Additional Information on the Data Classification

S2.1 Annelation Pattern Classification

In this section, we provide further information regarding the classification of the various pyrene-based PBHs according to the annelation patterns.

- There are eight annelation patterns in total: **a**, **b**, **aa**, **bb**, **ab**, **aab**, **abb**, **aabb**. Any additional annelations would result in *peri*-condensed PBHs, which are outside the scope of the current investigation.

- Molecules are classified according to the highest number of annelated positions. For example, a molecule that has two *a*-annelations and two *b*-annelations will be classified as **aabb**, not **a**, **b**, **aa**, **bb**, **ab**, **aab**, or **abb**, even though those are in principle contained within the molecule. A molecule with one *a*-annelation and one *b*-annelation will be classified as **ab**, not **a** or **b**. This guarantees that each molecule can only belong to one pattern group.

- In order to satisfy the different annelation patterns, molecules must contain a minimal number of rings. For example, molecules belonging to **a** and **b** must have at least five rings; molecules belonging to **aab** and **abb** must have at least seven rings.

- Molecules with the same annelation pattern can have varying numbers of rings (see Figure S3). All of the 'additional' rings (i.e., beyond the minimal number) will not be annelated directly on the pyrene core.

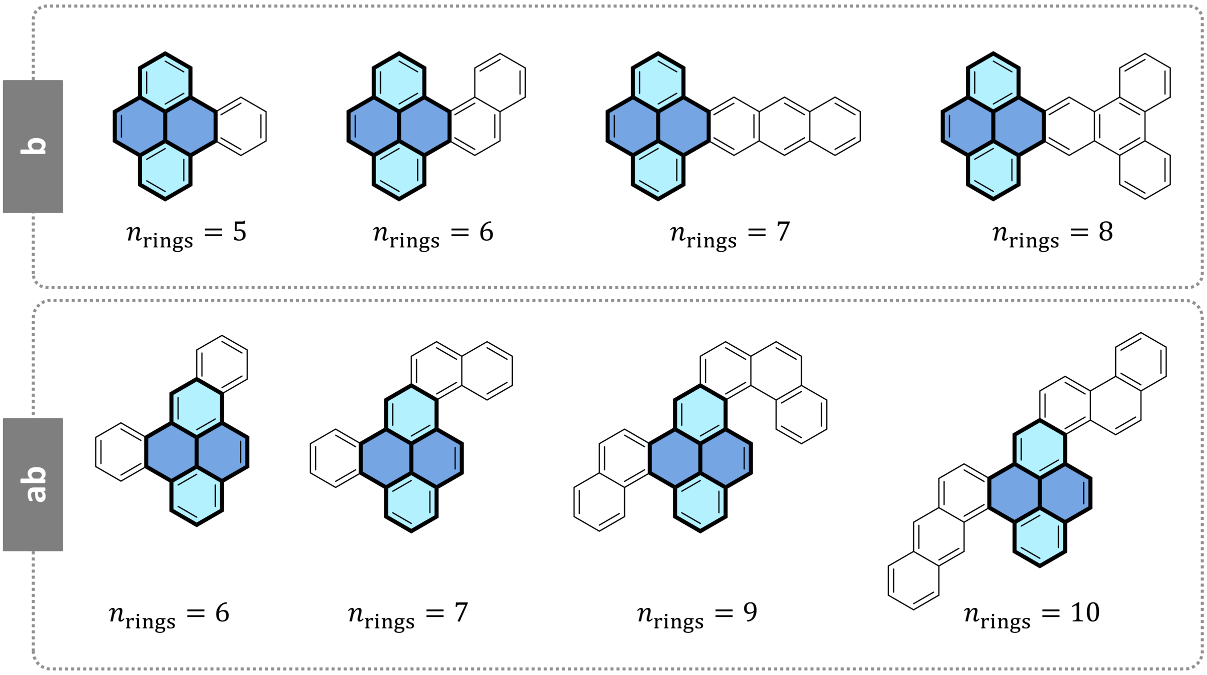

Figure S3. Examples of different-sized molecules belonging to a specific annelation pattern.

S2.2 Data Overview

Table S5 provides the number of isomers belonging to each of the pattern types described in the main text.

Table S5. Number of molecules of each annelation pattern.

Pattern	Sub-Pattern	No. Molecules	Sum
**a**	a^1	724	724
**aa**	aa^1	383	768
	aa^2	385	
**aab**	aab^1	190	760
	aab^2	373	
	aab^3	197	
**aabb**	$aabb^1$	57	113
	$aabb^2$	56	
**ab**	ab^1	720	1474
	ab^2	754	
**abb**	abb^1	364	364
**b**	b^1	359	359
**bb**	bb^1	204	204

Tables S6-S9 contain the statistical description of the data set grouped by the chemical formula, the pattern, the sub-patterns, and the ΔClar values, respectively.

Table S6. Statistical description of the data set grouped by chemical formula.

		HOMO (eV)		LUMO (eV)		$E_{\mathrm{H-L}}$ (eV)		aIP (eV)		aEA (eV)		E_{rel} (kcal/mol)	
formula	count	mean	std	mean	std	mean	std	mean	std	mean	std	mean	std
$C_{20}H_{12}$	2	-6.7534	0.2510	-0.8476	0.2384	5.9057	0.4894	7.0609	0.2596	-0.5738	0.2484	1.6983	2.4018
$C_{24}H_{14}$	10	-6.6355	0.2371	-0.9979	0.2168	5.6376	0.4513	6.8817	0.2452	-0.7757	0.2285	5.3224	3.2970
$C_{28}H_{16}$	39	-6.5464	0.1852	-1.0971	0.1923	5.4492	0.3744	6.7456	0.1909	-0.9203	0.1926	8.2203	4.3092
$C_{32}H_{18}$	184	-6.4939	0.1849	-1.1645	0.1959	5.3295	0.3778	6.6573	0.1887	-1.0176	0.1952	12.0243	5.1430
$C_{36}H_{20}$	819	-6.4564	0.1773	-1.2107	0.1918	5.2457	0.3659	6.5924	0.1791	-1.0898	0.1890	14.6560	5.8397
$C_{40}H_{22}$	3712	-6.4272	0.1740	-1.2475	0.1892	5.1797	0.3595	6.5411	0.1764	-1.1458	0.1855	16.6618	6.3913

Table S7. Statistical description of the data set grouped by Pattern.

		HOMO (eV)		LUMO (eV)		$E_{\mathrm{H-L}}$ (eV)		aIP (eV)		aEA (eV)		E_{rel} (kcal/mol)	
Pattern	count	mean	std	mean	std	mean	std	mean	std	mean	std	mean	std
**aa**	768	-6.2960	0.1361	-1.3697	0.1657	4.9263	0.2996	6.4034	0.1303	-1.2722	0.1537	14.8492	4.9809
**aab**	760	-6.3615	0.1447	-1.3080	0.1604	5.0535	0.3021	6.4678	0.1437	-1.2130	0.1525	18.5834	6.7876
**a**	724	-6.4260	0.1423	-1.2390	0.1756	5.1870	0.3142	6.5647	0.1406	-1.1161	0.1686	14.5702	5.3963
**aabb**	113	-6.4530	0.1342	-1.2244	0.1374	5.2286	0.2684	6.5637	0.1369	-1.1265	0.1322	22.1685	4.4199
**ab**	1474	-6.4623	0.1535	-1.2130	0.1702	5.2492	0.3197	6.5822	0.1527	-1.1057	0.1642	15.9685	6.5480
**abb**	364	-6.5253	0.1493	-1.1539	0.1531	5.3714	0.2988	6.6324	0.1452	-1.0581	0.1421	16.7125	5.3783
**b**	359	-6.5900	0.1747	-1.0940	0.1986	5.4960	0.3686	6.7398	0.1840	-0.9675	0.2107	15.2994	7.7977
**bb**	204	-6.6547	0.1789	-1.0239	0.1962	5.6308	0.3677	6.7980	0.1824	-0.8879	0.1955	13.4643	6.6037

Table S8. Statistical description of the data set grouped by Sub-Pattern.

		HOMO (eV)		LUMO (eV)		$E_{\mathrm{H-L}}$ (eV)		aIP (eV)		aEA (eV)		E_{rel} (kcal/mol)	
Sub-Pattern	count	mean	std	mean	std	mean	std	mean	std	mean	std	mean	std
a^1	724	-6.4260	0.1423	-1.2390	0.1756	5.1870	0.3142	6.5647	0.1406	-1.1161	0.1686	14.5702	5.3963
aa^1	383	-6.2452	0.1439	-1.4338	0.1657	4.8113	0.3075	6.3598	0.1377	-1.3265	0.1566	16.0114	4.8458
aa^2	385	-6.3466	0.1061	-1.3060	0.1391	5.0407	0.2427	6.4468	0.1062	-1.2182	0.1300	13.6931	4.8485
aab^1	190	-6.3739	0.1196	-1.2823	0.1320	5.0916	0.2487	6.4788	0.1227	-1.1917	0.1275	25.0784	4.3915
aab^2	373	-6.3097	0.1464	-1.3749	0.1534	4.9348	0.2978	6.4199	0.1438	-1.2744	0.1463	19.0815	4.8165
aab^3	197	-6.4477	0.1180	-1.2062	0.1363	5.2415	0.2485	6.5479	0.1238	-1.1174	0.1304	11.3762	4.7129
$aabb^1$	57	-6.5032	0.1150	-1.1699	0.1191	5.3334	0.2296	6.6092	0.1237	-1.0774	0.1171	22.7712	4.4498
$aabb^2$	56	-6.4019	0.1339	-1.2799	0.1333	5.1219	0.2647	6.5174	0.1351	-1.1764	0.1289	21.5551	4.3429
ab^1	720	-6.4412	0.1545	-1.2384	0.1660	5.2028	0.3174	6.5571	0.1522	-1.1366	0.1577	18.6431	5.9817
ab^2	754	-6.4824	0.1499	-1.1888	0.1707	5.2936	0.3156	6.6063	0.1494	-1.0762	0.1649	13.4146	6.0280
abb^1	364	-6.5253	0.1493	-1.1539	0.1531	5.3714	0.2988	6.6324	0.1452	-1.0581	0.1421	16.7125	5.3783
b^1	359	-6.5900	0.1747	-1.0940	0.1986	5.4960	0.3686	6.7398	0.1840	-0.9675	0.2107	15.2994	7.7977
bb^1	204	-6.6547	0.1789	-1.0239	0.1962	5.6308	0.3677	6.7980	0.1824	-0.8879	0.1955	13.4643	6.6037

Table S9. Statistical description of the data set grouped by ΔClar.

		HOMO (eV)		LUMO (eV)		$E_{\mathrm{H-L}}$ (eV)		aIP (eV)		aEA (eV)		E_{rel} (kcal/mol)	
ΔClar	count	mean	std	mean	std	mean	std	mean	std	mean	std	mean	std
-2	768	-6.2960	0.1361	-1.3697	0.1657	4.9263	0.2996	6.4034	0.1303	-1.2722	0.1537	14.8492	4.9809
-1	760	-6.3615	0.1447	-1.3080	0.1604	5.0535	0.3021	6.4678	0.1437	-1.2130	0.1525	18.5834	6.7876
0	837	-6.4296	0.1415	-1.2370	0.1709	5.1926	0.3086	6.5646	0.1400	-1.1175	0.1642	15.5960	5.8781
1	1474	-6.4623	0.1535	-1.2130	0.1702	5.2492	0.3197	6.5822	0.1527	-1.1057	0.1642	15.9685	6.5480
2	364	-6.5253	0.1493	-1.1539	0.1531	5.3714	0.2988	6.6324	0.1452	-1.0581	0.1421	16.7125	5.3783
3	359	-6.5900	0.1747	-1.0940	0.1986	5.4960	0.3686	6.7398	0.1840	-0.9675	0.2107	15.2994	7.7977
4	204	-6.6547	0.1789	-1.0239	0.1962	5.6308	0.3677	6.7980	0.1824	-0.8879	0.1955	13.4643	6.6037

S3 Size Dependency of Properties and Defined Features

In the main text, we describe the trends of six molecular properties and several newly defined features. In this section, we show their dependence on molecular size.

S3.1 Molecular Properties

It is well-known that changing the size of a conjugated system affects the molecular orbital energies of the molecule. As the conjugated system is increased, this tends to push the HOMO energy up and the LUMO energy down, decreasing the $\Delta E_{\mathrm{H-L}}$. The ionization potential and electron affinity are also affected, because the energy of removing or adding an electron is dependent upon the energy of the orbital from/to which the electron is taken/added. Additionally, charged systems are stabilized by delocalization, meaning that larger systems can more easily stabilize additional charge. Finally, the relative energy (i.e., thermodynamic stability) of different molecules is dependent on size for all the aforementioned reasons, but also because additional rings allow for more opportunities to form non-planar motifs, such as coves, fjords, and helices.

Due to this expected size dependency, it is important to rule out size effects as the reason for the observed trends, rather than from the structure-property relationships that we outline in the text. To verify that this is indeed the case, we plotted the distributions of the individual properties (similar to Figure 2 in the main text), this time also separating the data according to the number of rings. We can see in Figure S4 that the trend does not change with the number of rings, thus ruling out the size effect.

Although the general trends are not changed by the inclusion of molecules with varying sizes, it is clear that there is a size-dependency that may obscure some of the subtler features of the observed trends. In particular, we found that ΔClar is an important parameter in determining the π-system properties. Therefore, we also compared the relationship between the properties and ΔClar for all data (FIG) and for just the molecules with the chemical formula

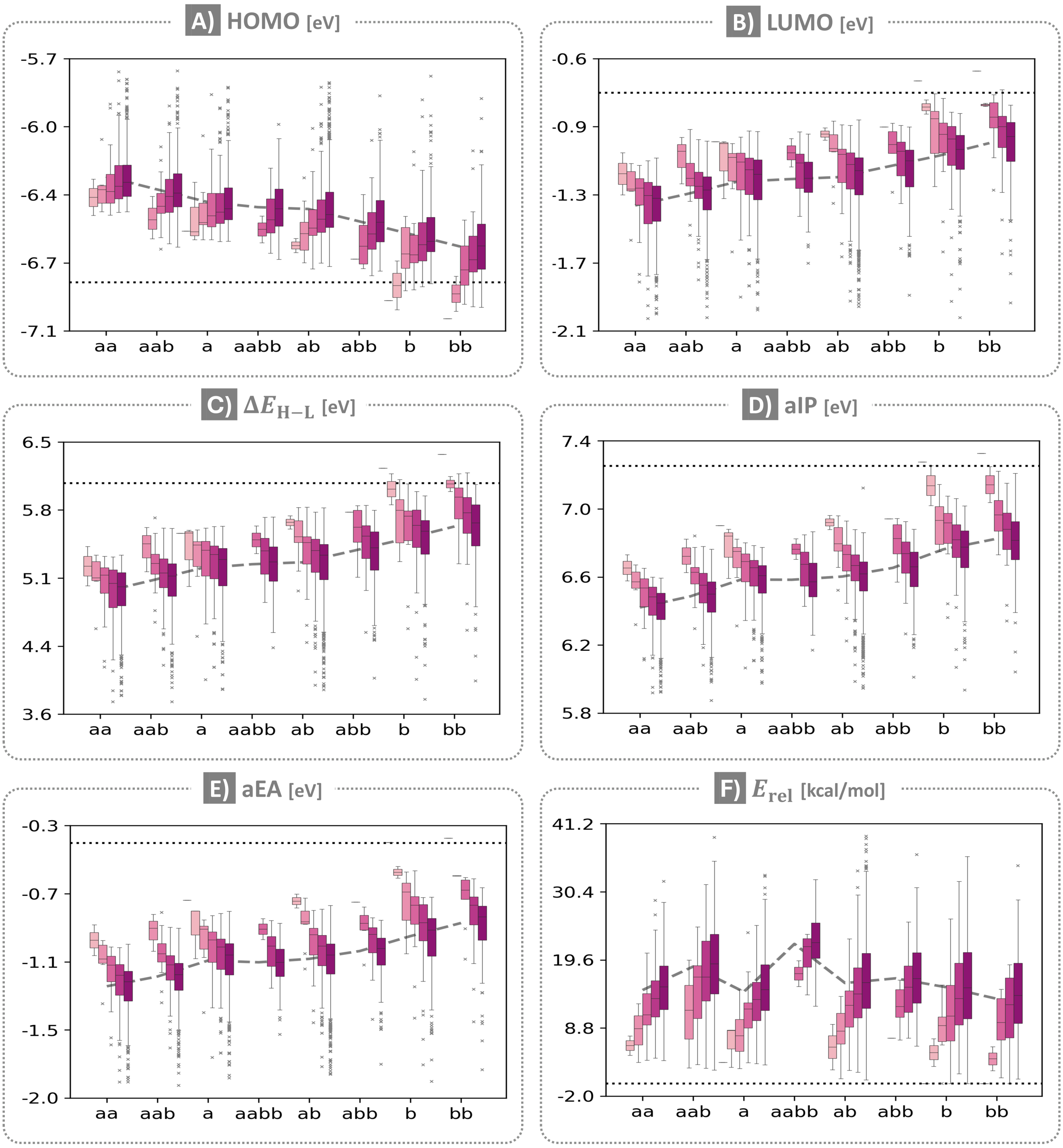

Figure S4. Box-and-whisker plots of the DFT-calculated values of the A) HOMO, B) LUMO, C) $\Delta E_{\mathrm{H-L}}$, D) aIP, E) aEA, and F) E_{rel}, broken down by annelation patterns and further separated by number of rings in the molecule. The dashed gray line denotes the average value, the dashed red line denotes the value of pyrene.

S3.2 Defined Features

In the main text, we define several features and examine their correlations to the molecular properties. Here, as well, we wish to demonstrate which of the features shows a dependency on molecular size, and which does not.

To this end, we generated stacked histogram plot, which show the breakdown of the different features and how many molecules of different sizes (as a percentage of the total dataset) belong to each of the feature values.

As can be seen from the plots below, the total number of Clar sextets in a molecule and the longest linear stretch in a molecule both increase as the molecular size increases. This is not surprising, as increasing the number of rings creates more opportunities for the formation of Clar sextets, as well as more opportunities to form linearly annelated substructures. In contrast, the ΔClar feature does not show a size-dependency. This is because this metric balances between fixed and migrating Clar sextets, which are dependent on the molecular geometry of a substructure and not the entire molecule.

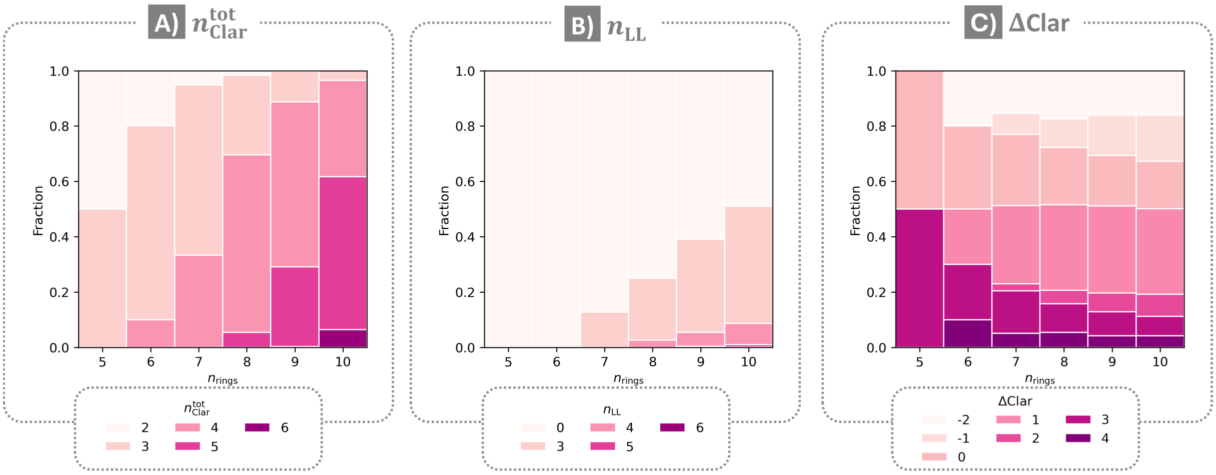

Figure S5. Stacked histograms of A) $n_{\text{Clar}}^{\text{tot}}$, B) n_{LL}, and C) ΔClar. Each block describes the percentage of molecules of a specific feature value, colored by the number of rings in the molecules.

S4 Additional ΔClar Analysis

In the main text, we discuss the relationship to the ΔClar metric, including a demonstration that the relationship has an additional size-dependent effect and is also intertwined with the presence of linear stretches.

Although the general trends are not changed, it is clear that these additional effects obscure some of the subtler features of the ΔClar relationship. Therefore, in this section we compare the relationship between the properties and ΔClar for all data (Figure S6) and the same relationships observed for just the molecules with the chemical formula $C_{40}H_{22}$ and n_{LL}=0 (Figure S7).

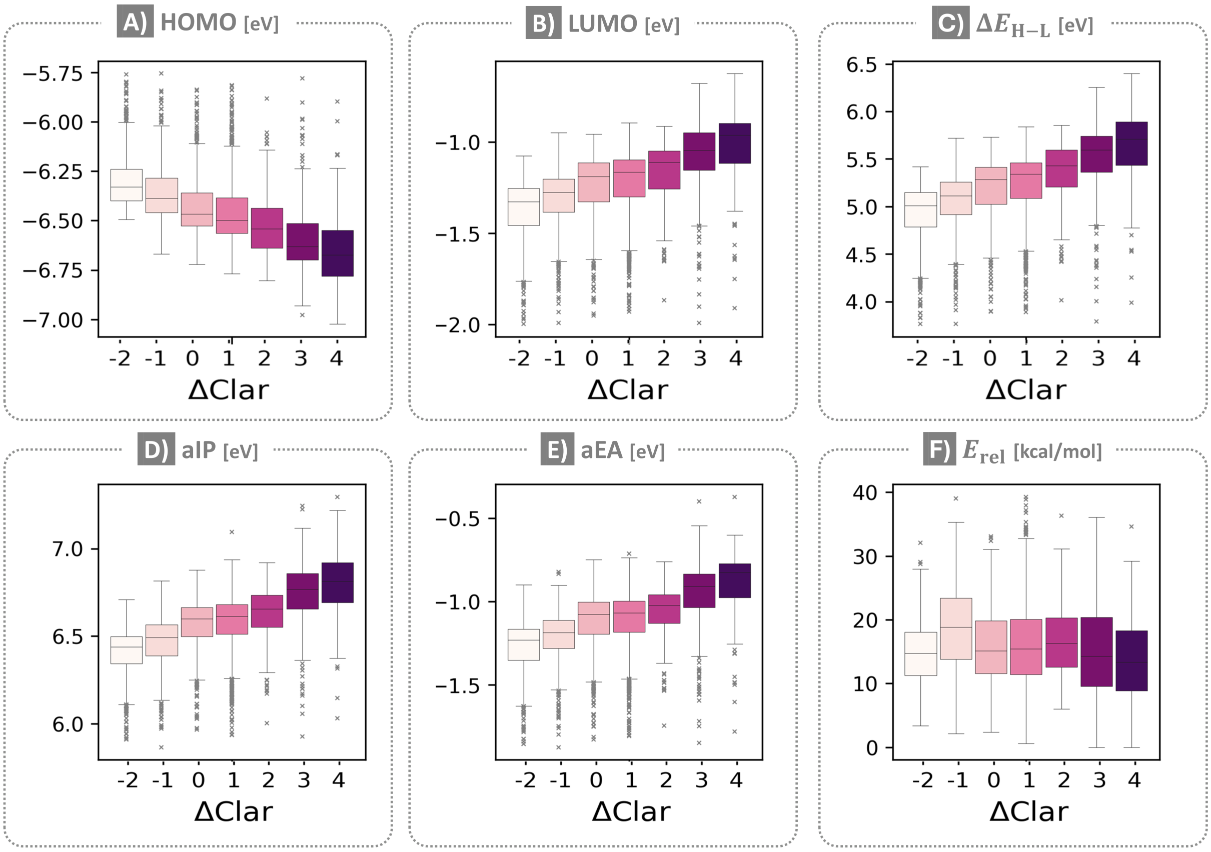

Figure S6. Box-and-whisker plots of the DFT-calculated values of the A) HOMO, B) LUMO, C) ΔE_{H-L}, D) aIP, E) aEA, and F) E_{rel}, separated by their ΔClar values. All data in the Pyrenes dataset included.

The comparison between the two sets of plots reveals a few interesting findings. First, we observe that the overlap between the groups decreases noticeably. Meaning, when looking at molecules of the same size and without the confounding effect of long linear stretches, the ΔClar metric becomes a more impactful descriptor and is therefore also a more powerful predictor of property value ranges. One can especially note the large gap between ΔClar=2 and ΔClar=3 ranges (essentially no overlap), which show a substantial shift in properties upon formation of a third Clar sextet. Second, we see that many of the outliers disappear. This is perhaps not overly surprising. We already saw in the main text that ΔClar annelation pattern is a good predictor of the *average* behavior. The present results simply corroborate that the outlying molecules are characterized by other structural features, most likely longer linear stretches.

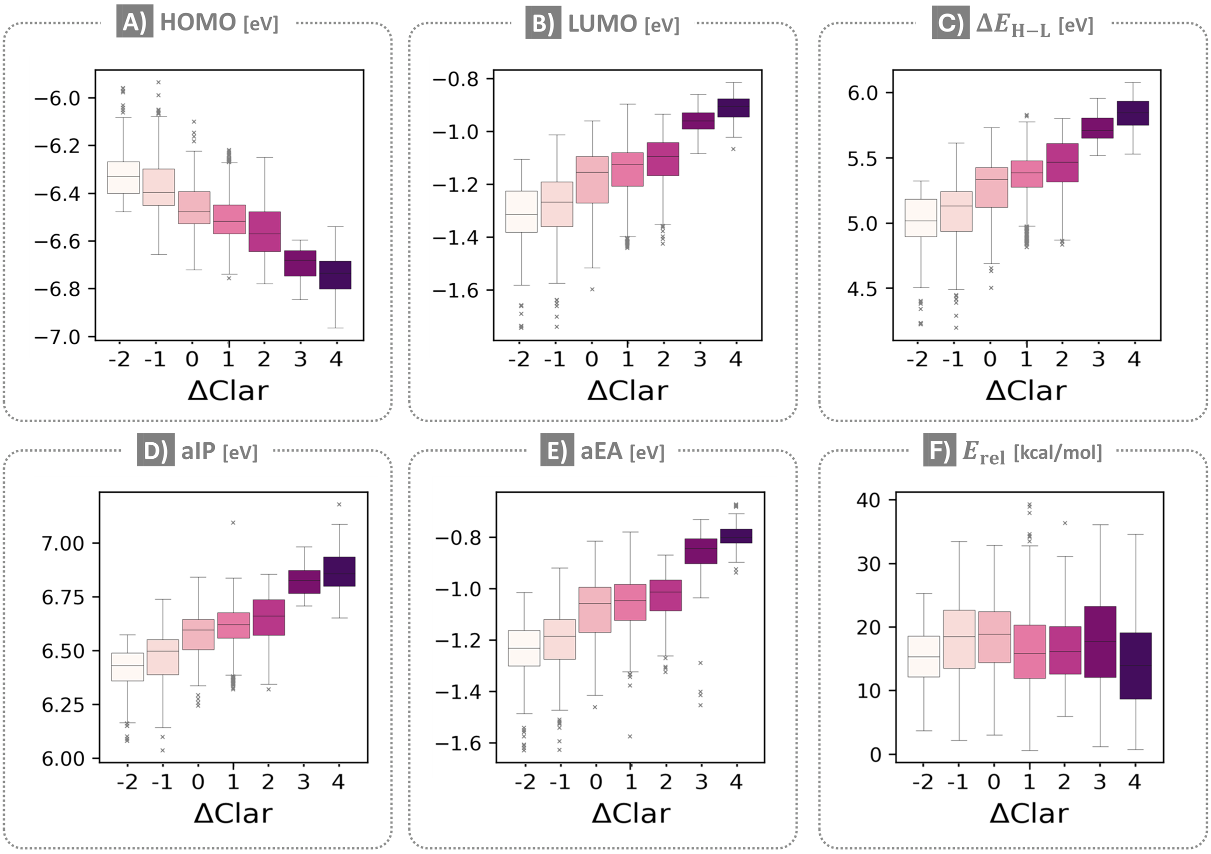

Figure S7. Box-and-whisker plots of the DFT-calculated values of the A) HOMO, B) LUMO, C) $\Delta E_{\mathrm{H-L}}$, D) aIP, E) aEA, and F) E_{rel}, separated by their ΔClar values. Only $C_{40}H_{22}$ molecules with n_{LL}=0 from the Pyrenes dataset included.

S5 Correlation of E_{strain} with Structure

Figure S8 below shows the correlation of E_{strain} with n_{coves} (A) and with the sum of cove and bay motifs ($n_{\mathrm{coves}} + n^{\mathrm{a}}_{\mathrm{bays}} + n^{\mathrm{b}}_{\mathrm{bays}}$, B). The correlation with n_{coves} is quite good, and substantiates our finding that coves contribute the most to strain. However, while it captures the main effect, it lacks the sensitivity to differentiate between the different a/b-motifs. Thus, we first tried to add the effect of the bay motifs, as seen in plot B. This had a deleterious effect on the correlation, because of the substantial overestimation of the effect of the bay motifs. As we show in the main text, the bay motifs have much smaller contributions than the cove motif.

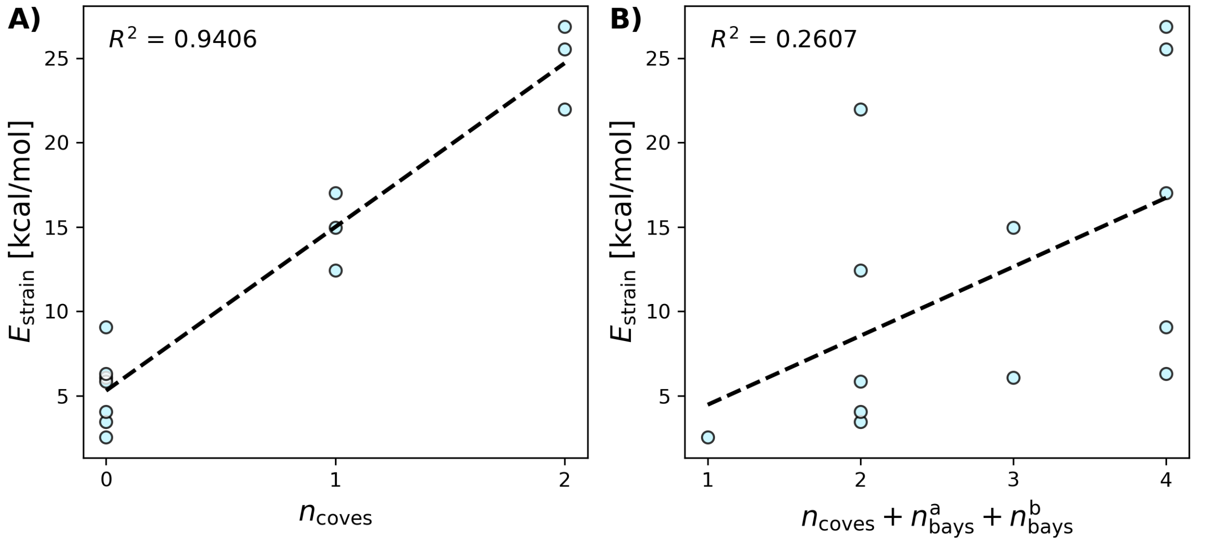

Figure S8. A) Scatter plot of E_{strain}vs n_{coves} and the corresponding regression line. B) Scatter plot of E_{strain} vs the sum of cove and bay motifs, $n_{\mathrm{coves}} + n^{\mathrm{a}}_{\mathrm{bays}} + n^{\mathrm{b}}_{\mathrm{bays}}$, and the corresponding regression line.

S6 Feature optimization to predict $E_{\text{rel}}^{\text{avg}}$

As discussed in the main text, we fit a linear equation using the features we designed, $n_{\text{strain}}^{\text{avg}}$ and ΔClar, to predict $E_{\text{rel}}^{\text{avg}}$. To find the optimal parameter fitting the following equation:

$$E_{\text{rel}}^{\text{avg}} = \alpha \cdot n_{\text{strain}}^{\text{avg}} + \beta \cdot \Delta\text{Clar} + \gamma \tag{S1}$$

we used the `optimize.curve_fit` function from the python package SciPy,[S19] version 1.15.3, to optimize the α, β, and γ parameters.

The following code shows how this was achieved.

```
import numpy as np
from scipy.optimize import curve_fit

def model(X, a, b, c):
    """
    Linear equation to fit
    """
    x1, x2 = X
    return a*x1 + b*x2 + c

# Prepare the data
# x1 and x2 are the features we want to fit to y
x1 = df_mean['n_strain_avg'].values # get the n_strain_avg values
x2 = df_mean['dClar'].values        # get the Delta Clar values
y = df_mean['erel_kcalmol'].values  # target, i.e., what we want to fit

# Stack x1 and x2 for curve_fit
X = np.vstack([x1, x2])

# Initial guess for parameters [a, b, c]
p0 = [1, 1, 0]

# Fit the curve
popt, pcov = curve_fit(model, X, y, p0=p0)

# Get the optimized parameters
a, b, c = popt
print(f"y = {a:.2f}*x1 + {b:.2f}*x2 + {c:.2f}")

# Calculated the predicted E_rel^avg using the optimal linear model
y_pred = model(X, *popt)
```

References

(S1) Neese, F. *Wiley Interdiscip. Rev. Comput. Mol. Sci.* **2012**, *2*, 73–78.

(S2) Neese, F. *Wiley Interdiscip. Rev. Comput. Mol.* **2022**, *12*, e1606.

(S3) Becke, A. D. *J. Chem. Phys* **1993**, *98*, 5648–5652.

(S4) Lee, C.; Yang, W.; Parr, R. G. *Phys. Rev. B* **1988**, *37*, 785–789.

(S5) Miehlich, B.; Savin, A.; Stoll, H.; Preuss, H. *Chem. Phys. Lett.* **1989**, *157*, 200–206.

(S6) Hertwig, R. H.; Koch, W. *Chem. Phys. Lett.* **1997**, *268*, 345–351.

(S7) Yanai, T.; Tew, D. P.; Handy, N. C. *Chem. Phys. Lett.* **2004**, *393*, 51–57.

(S8) Weigend, F.; Ahlrichs, R. *Phys. Chem. Chem. Phys.* **2005**, *7*, 3297.

(S9) Dunning, T. H. *J. Chem. Phys.* **1989**, *90*, 1007–1023.

(S10) Kendall, R. A.; Dunning, T. H.; Harrison, R. J. *J. Chem. Phys.* **1992**, *96*, 6796–6806.

(S11) Woon, D. E.; Dunning, T. H. *J. Chem. Phys.* **1993**, *98*, 1358–1371.

(S12) Grimme, S.; Antony, J.; Ehrlich, S.; Krieg, H. *J. Chem. Phys* **2010**, *132*, 154104.

(S13) Johnson, E. R.; Becke, A. D. *J. Chem. Phys* **2005**, *123*, 024101.

(S14) Grimme, S.; Ehrlich, S.; Goerigk, L. *J. Comput. Chem.* **2011**, *32*, 1456–1465.

(S15) Liang, J.; Feng, X.; Hait, D.; Head-Gordon, M. *J. Chem. Theory Comput.* **2022-06**, *18*, 3460–3473.

(S16) Wahab, A.; Gershoni-Poranne, R. *Phys. Chem. Chem. Phys.* **2024**, *26*, 15344–15357.

(S17) Woon, D. E.; Dunning Jr., T. H. *J. Chem. Phys.* **1994**, *100*, 2975–2988.

(S18) Rappoport, D.; Furche, F. *J. Chem. Phys.* **2010**, *133*, 134105.

(S19) Virtanen, P. et al. *Nature Methods* **2020**, *17*, 261–272.
